# The transcriptome of *Pinus pinaster* under *Fusarium circinatum* challenge

**DOI:** 10.1186/s12864-019-6444-0

**Published:** 2020-01-08

**Authors:** Laura Hernandez-Escribano, Erik A. Visser, Eugenia Iturritxa, Rosa Raposo, Sanushka Naidoo

**Affiliations:** 1 Instituto Nacional de Investigación y Tecnología Agraria y Alimentaria, Centro de Investigación Forestal (INIA-CIFOR), Madrid, Spain; 2 Departamento de Biotecnología-Biología Vegetal, Escuela Técnica Superior de Ingeniería Agronómica, Alimentaria y de Biosistemas, Universidad Politécnica de Madrid, Madrid, Spain; 3 Department of Biochemistry, Genetics and Microbiology, Forestry and Agricultural Biotechnology Institute (FABI), Centre for Bioinformatics and Computational Biology, University of Pretoria, Pretoria, South Africa; 4 NEIKER, Granja Modelo de Arkaute, Apdo 46, 01080 Vitoria-Gasteiz, Spain; 5 Instituto de Gestión Forestal Sostenible (iuFOR), Universidad de Valladolid/INIA, Valladolid, Spain

**Keywords:** *Pinus pinaster*, Salicylic acid, Jasmonic acid, *Fusarium circinatum*, Fungal hormone production, Defense response, de novo transcriptome assembly, Dual RNAseq

## Abstract

**Background:**

*Fusarium circinatum*, the causal agent of pitch canker disease, poses a serious threat to several *Pinus* species affecting plantations and nurseries. Although *Pinus pinaster* has shown moderate resistance to *F. circinatum*, the molecular mechanisms of defense in this host are still unknown. Phytohormones produced by the plant and by the pathogen are known to play a crucial role in determining the outcome of plant-pathogen interactions. Therefore, the aim of this study was to determine the role of phytohormones in *F. circinatum* virulence, that compromise host resistance.

**Results:**

A high quality *P. pinaster* de novo transcriptome assembly was generated, represented by 24,375 sequences from which 17,593 were full length genes, and utilized to determine the expression profiles of both organisms during the infection process at 3, 5 and 10 days post-inoculation using a dual RNA-sequencing approach. The moderate resistance shown by *Pinus pinaster* at the early time points may be explained by the expression profiles pertaining to early recognition of the pathogen, the induction of pathogenesis-related proteins and the activation of complex phytohormone signaling pathways that involves crosstalk between salicylic acid, jasmonic acid, ethylene and possibly auxins. Moreover, the expression of *F. circinatum* genes related to hormone biosynthesis suggests manipulation of the host phytohormone balance to its own benefit.

**Conclusions:**

We hypothesize three key steps of host manipulation: perturbing ethylene homeostasis by fungal expression of genes related to ethylene biosynthesis, blocking jasmonic acid signaling by coronatine insensitive 1 (COI1) suppression, and preventing salicylic acid biosynthesis from the chorismate pathway by the synthesis of isochorismatase family hydrolase (ICSH) genes. These results warrant further testing in *F. circinatum* mutants to confirm the mechanism behind perturbing host phytohormone homeostasis.

## Background

Plant hormones play a crucial role in plant-pathogen interactions, especially salicylic acid (SA), jasmonic acid (JA) and ethylene (ET) which are primary signals for induction of defense response. Generally, ET and JA act synergistically in response to the infection of necrotrophic fungi, while SA is most commonly expressed in response to biotrophic or hemibiotrophic fungi [41]. However, this is only a simplistic classification and crosstalk between phytohormones are much more complex and context dependent. Although an antagonistic relation between JA and SA has been reported [59, 76, 95], this antagonism seems to be absent in the defense response of *Arabidopsis* to *Plectosphaerella cucumerina* [8], *Pseudomonas syringae* and *Peronospora parasitica* [23]. A cooperation between the two phytohormones has also been described in *Picea abies* [4] and *Zea mays* [36]. A role of auxins in plant-pathogen interaction has also been reported, modulating signaling pathways of other hormones, resulting in positive or negative effect in resistance [5, 58, 73, 77]. Considerable effort has been dedicated to understanding phytohormone signaling in plant defense, while knowledge on the role of fungal hormone production is limited.

A remarkable aspect of *Fusarium* species in the *Fusarium fujikuroi* species complex (FFC), is the ability to synthesize phytohormones, including gibberellins [12, 101] and auxins [103], that contribute to plant disease. However, the underlying molecular mechanism as well as their role in plant interactions remains unclear. Two mechanisms have been suggested: perturbing plant processes to favor invasion and nutrient uptake, and/or acting as signals for the fungus to engage appropriate physiological processes to allow adaption to the new environment [21]. Gibberellic acid (GA) production has been well described in the rice-infecting fungus *Fusarium fujikuroi* [12] and a correlation between GA levels and virulence has been reported [29]. GA biosynthetic genes are organized in a gene cluster, and while most of the species of the *F. fujikuroi* species complex have the entire GA biosynthetic gene cluster, *F. circinatum* was reported to have only one gene [12, 64]. Indol-3-acetic acid (IAA), the most common form of auxins, can be synthesized from tryptophan by the indol-3-acetamide (IAM) pathway, and *IAM*-related genes have been reported in four *Fusarium* pathogenic fungi: *Fusarium verticillioides*, *Fusarium oxysporum*, *F. fujikuroi* and *Fusarium proliferatum* [103]. In the same study, the deletion of an *IAM*-related gene resulted in drastic reduction of IAA production in *F. proliferatum*. Similarly, *F. oxysporum* transgenic lines containing two *IAM* genes produced significantly more IAA than the wild type when infecting Orobanche, leading to enhanced virulence [24]. ET producing fungi range from necrotrophic, like *Botrytis cinerea*, to biotrophic, such as *F. oxysoporum* f. sp. *pini*, and a role in perturbing the plant phytohormone homeostasis has been suggested [21]. Silicon treatment has been shown to induce brown spot resistance in rice against *Cochliobolus miyabeanus* by disarming fungal ET [105]. In the case of *Colletotrichum* sp. pathogens, ET is required for the formation of appressoria [38]. To our knowledge, expression of *F. circinatum* genes related to hormone biosynthesis or signaling, besides GA, has not been studied.

*Fusarium circinatum* Nirenberg & O’Donnell is described as one of the most important pathogens worldwide, affecting more than 60 *Pinus* species as well as *Pseudotsuga menziesii* Mirb. (Franco) [118]. The fungus can cause damage to seedlings as a wilt disease but also to mature trees, known as pitch canker disease. In Europe, the pathogen is currently present in the Atlantic area of Spain [87] and in Portugal [14], where *Pinus* species are grown. The maritime pine (*Pinus pinaster* Ait.) is the dominant species in the Mediterranean area with more than 2.3 million hectares [84], especially in the Atlantic coast of France, Portugal and Spain. Although *P. pinaster* has shown moderate resistance to the pathogen (mean lesion length of 5 mm compared to 28 mm in *P. radiata* [49] and a 66% incidence rate in a provenance/progeny trial of artificially inoculated seedlings [35], the presence of the fungus in the area represents a serious threat to this species. In spite of the special effort done in management of pitch canker disease based on cultural, biochemical and biological control, the pathogen has still not been eradicated. Therefore, selection of genetically resistant genotypes against *F. circinatum* seems to be an appropriate approach for disease management.

Advances in sequencing approaches have allowed the generation of new resources for conifers. RNA sequencing (RNAseq) approach allows the characterization of the transcriptome even in species for which no reference genome is available or is incomplete. In both instances, RNAseq reads can be assembled de novo into a transcriptome [17, 83, 110]. A variant of this technology is the dual RNAseq, which captures the transcriptome of the host and pathogen simultaneously, so that gene expression in both organisms may be determined [45, 66, 72, 116].

Various transcriptional changes are apparent in the *Fusarium circinatum*-*Pinus* pathosystem. Transcriptome analysis of *Pinus radiata* inoculated seedlings was recently published [18] showing induction of genes related to abscisic acid (ABA) signaling, auxin responsive-like proteins, gibberellin-regulated protein precursor, as well as induction of pathogenesis-related (PR) proteins, phosphorylase family protein (PFP) and genes related to physical and chemical barriers to restrict pathogen invasion. Similarly, Donoso et al., [32] detected an up-regulation of genes encoding thaumatin-like protein (PR5) in *P. radiata* inoculated seedlings by a RT-qPCR assay. Davis et al., [27] reported SA and JA induction of chitinase (PR3) in pine seedlings inoculated with *Fusarium subglutinans* f. sp. *pini*, suggesting a potential role for PR proteins in pine defense.

While most pine studies have focused their attention on host defense [18, 32], little is known about *F. circinatum* genes involved in pathogenicity. Muñoz-Adalia et al. [70] suggested 5 candidate genes that could be involved in *F. circinatum* virulence based on high similarity with other *Fusarium* species. Recently, using a dual RNAseq approach, Visser et al., [109] reported differences in the expression of *F. circinatum* ergosterol biosynthesis genes during the infection of *Pinus tecunumanii* and *Pinus patula* seedlings, suggesting a role for this pathway in pathogen virulence. The study also alluded to a role of phytohormone signaling in pine defense.

The aim of this study was to elucidate the role of phytohormones in moderate resistance of *P. pinaster* to *F. circinatum* and determine key steps where the pathogen could be manipulating host phytohormone balance to its own benefit, leading to host susceptibility. For this purpose, we determined by a dual RNAseq approach the expression profile of both organisms during the host-pathogen interaction at different times after inoculation (3, 5 and 10 days post-inoculation, dpi). Furthermore, a good quality *P. pinaster* de novo transcriptome assembly was generated, improving current *P. pinaster* genetic resources.

## Results

### Pathogen colonization and symptom development

**Fig. 1:**
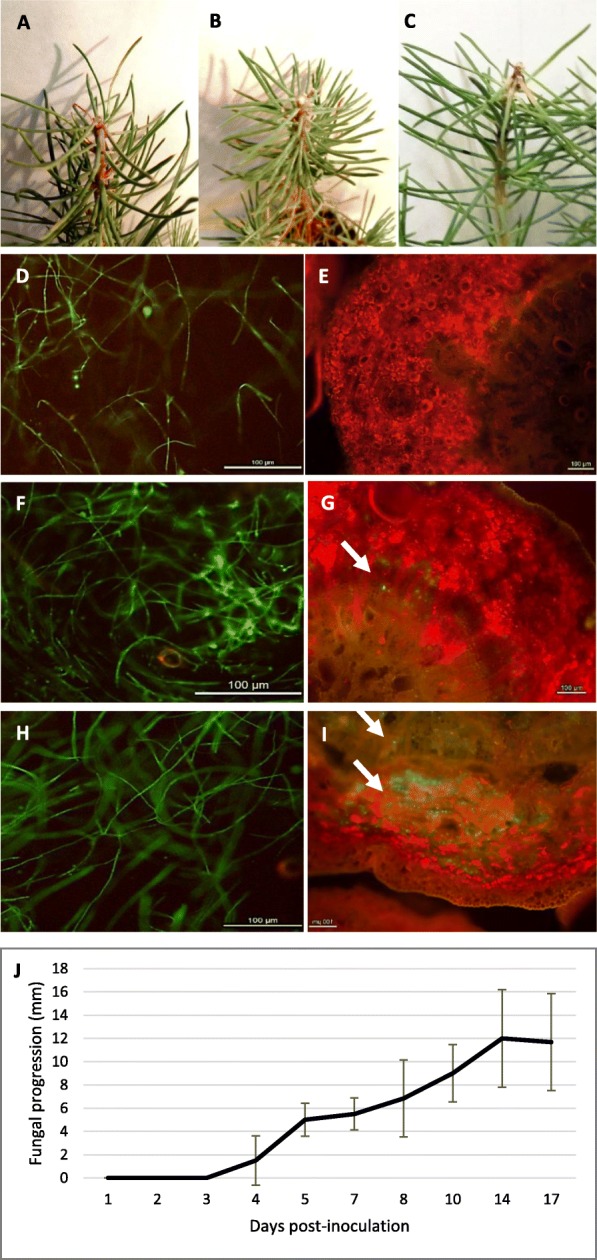
*Fusarium circinatum* inoculation of *Pinus pinaster*. **a**-**c**: symptoms at the shoot tip of inoculated *Pinus pinaster* seedlings at each sampling time point. **a**: 3 dpi, no visible symptoms. **b**: 5 dpi, no visible symptoms. **c**: 10 dpi, symptoms at the inoculation site. **e**, **g**, **i**: transverse sections of *P. pinaster* inoculated shoot tip visualized under epifluorescence microscope at each sampling time after inoculation. **d**, **f**, **h**: growth of the fungus in the resin drop at each time point. **d**-**e**: 3 dpi. **f**-**g**: 5 dpi. **h**-**i**: 10 dpi. White arrows indicate colonization of the fungus within host tissue. Bar 100 μm. **j**: progression of a green fluorescent protein (GFP)-tagged *Fusarium circinatum* strain within *Pinus pinaster* shoot tissue at different days after inoculation

*Fusarium circinatum* was observed growing in the resin drop of *P. pinaster* seedlings during the first three days. At 4 dpi the pathogen had entered host tissue in only one out of six plants analyzed while at 5 dpi, the pathogen had penetrated all of them. During the following days, the fungus continued growing within the host and progressed from the tip along the stem. At 8 dpi some of the plants showed visible damage at the inoculation site. At 11 dpi inoculated seedlings had a lesion length of approximately 5 mm from the tip. The sampling times chosen for the RNAseq assay were: 3 dpi (the fungus had not penetrated within the host and was growing in the resin drop), 5 dpi (the fungus penetrated within host tissue) and 10 dpi (lesion was visible in the shoot tip of all seedlings). Figure 1 shows example images from these time points and the progression of the fungus over the different time points.

By the end of the experiment, all inoculated seedlings showed symptoms of disease including discolored brown stems, necrosis and needle desiccation at the tip (Additional file 1). Inoculated seedlings showed a lesion length at the tip of 1.5 cm ± 0.59 (SD) and *F. circinatum* was re-isolated from all 6 tips cultured on Fusarium Specific Media. Mock-inoculated seedlings did not show symptoms of disease and the fungus was not recovered from any of them.

### *Pinus pinaster* de novo reference transcriptome

#### Quality of preliminary assemblies

A total of 21 different Trinity and TransABySS preliminary assemblies were built, showing differences in quality based on the parameters used (Additional file 2). None of the assemblies produced transcripts with unknown bases and the GC content were similar between them. In silico normalization of Trinity assemblies produced the highest number of transcripts, mean length and better N50 values. However, in non-normalized assemblies more fragments were mapped and the percentage of good mapped contigs was higher (Additional file 3). Therefore, all Trinity assemblies (normalized and non-normalized) were used for building the final de novo transcriptome assembly. When comparing Trinity and TransABySS assemblies at the same kmer value, Trinity showed better quality statistics, therefore TransABySS assemblies with the same kmer value as Trinity were discarded. As Trinity does not allow the use of kmer values higher than 32, all TransABySS assemblies with higher kmer values were conserved.

A total of 18 good quality preliminary assemblies were used as input for the EviGene pipeline to build the *P. pinaster* de novo transcriptome. When merging best quality assemblies, EviGene pipeline classified 90.7% of the sequences as redundant and uninformative, which means that 49,624 sequences (9.3%) were non redundant coding genes and were used to generate the *P. pinaster* transcriptome (Additional file 2).

Benchmarking Universal Single Copy Ortholog (BUSCO) analysis against the embryophyta_odb9 lineage database identified 1261 (87.57%) complete BUSCOs in a total of 1440 BUSCO groups searched, from which 1109 (77.01%) were single copy, 152 (10.56%) duplicated, 145 (10.07%) missing and 34 (2.36%) fragmented. Regarding the eukaryote_odb9 database, 294 completed (97.03%) BUSCOs were identified out of 303 groups searched, and 213 (70.30%) were single copy, 81 (26.73%) were duplicated, 7 (2.31%) were missing and only 2 (0.66%) fragmented (Additional file 4).

#### Annotation

Coding regions were predicted for 46,576 sequences with GeneMarkS-T. Of the aligned sequences, 29.15% (8584) were classified as non-pine origin contigs, mainly belonging to the *Fusarium* genera (*Fusarium mangiferae*, *F. fujikuroi*, *F. proliferatum*, *Fusarium nygamai*, *F. oxysporum*). After filtering contaminants, non-frame selected and unannotated sequences, the final *P. pinaster* de novo transcriptome assembly contained 24,375 sequences, from which 17,593 (72.18%) were full-length genes. Best hit selection of BLAST alignment against the 4 databases generated a total of 20,864 (85.60%) unique sequences, from which 9957 (40.85%) were informative. EggNOG annotation predicted 23,674 sequences with family assignment, 5425 with at least one pathway (KEGG) assignment and 22,863 predicted protein domains. EggNOG associated 7614, 11,096 and 5050 gene ontology (GO) terms to biological process (BP), cellular compartment (CC) and molecular function (MF) categories, respectively. InterProScan predicted 20,188 protein domains and associated 6293, 1929 and 9346 GO terms to BP, CC and MF category, respectively (Additional file 5).

GhostKOALA assigned 12,741 K numbers (of 35,090) mainly classified according to the KEGG Orthology System in the functional categories genetic information processing, environmental information processing and cellular processes. Mercator assigned functional annotation to 15,347 *P. pinaster* transcripts (Additional file 6).

### Mapping to the host and pathogen reference transcriptomes

Kallisto mapped a total of 964 million reads to the *F. circinatum* and *P. pinaster* combined dataset, which means 74.93% of the reads were mapped. In total, 72.97% of the reads were mapped to the *P. pinaster* de novo transcriptome reference, with similar mapping percentage between time points and between inoculated and mock-inoculated samples. Less than 8000 (< 0.01%) reads mapped to the *F. circinatum* reference transcriptome from any single mock-inoculated sample. For inoculated samples, 1.95% of the reads mapped to *F. circinatum* and an increase in the number of mapped reads was observed at the later time points owing to the growth of the pathogen (Additional file 7).

The principal component analysis (PCA) for *P. pinaster* and *F. circinatum* rlog data indicated clear separation of inoculated and mock-inoculated samples. The replicate samples show a high similarity with respect to the first two principal components for each time point. A small within group variance and a good separation of groups reflects the good quality of the analysis (Additional file 8).

### Host and pathogen DE genes

**Fig. 2:**
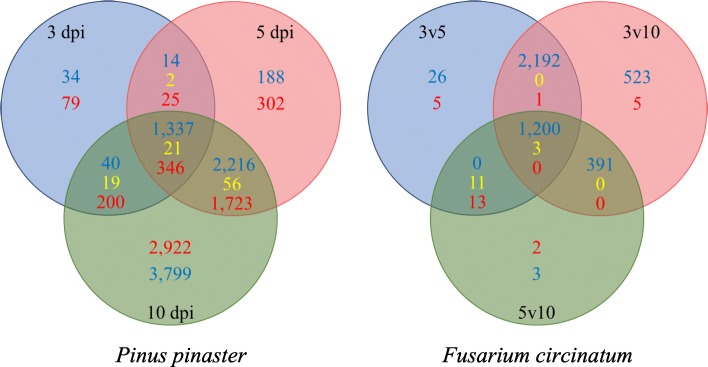
Venn’s diagram showing the overlap between differentially expressed (DE) genes. Left - *Pinus pinaster* DE genes at 3, 5 and 10 days post inoculation (dpi) in inoculated relative to mock-inoculated samples. Right – *Fusarium circinatum* DE genes between time points in inoculated samples. Red numbers – up-regulated genes, blue numbers – down-regulated genes, yellow numbers – genes contra-regulated between compared groups. 3v5: 3 relative to 5 dpi; 3v10: 3 relative to 10 dpi; 5v10: 5 relative to 10 dpi

A total of 13,323 differentially expressed (DE) genes were identified in inoculated *P. pinaster* samples compared to mock-inoculated ones. A notable increase of DE genes from 3 to 10 dpi was observed (Fig. 2). An unknown resistance protein gene was highly up-regulated at all time points and was the most up-regulated gene, with a log_2_ (Fold Change) of 25.08, 34.81 and 23.50 at 3, 5 and 10 dpi, respectively.

For *F. circinatum,* at 3, 5 and 10 dpi 93.17% (4070 genes), 99.8% (4366 genes) and 99.9% (4372 genes) of the DE genes were considered high confident (HC) expressed genes, respectively (Additional file 7). When comparing DE genes at 3 versus 5 dpi in inoculated seedlings, 3427 genes were down-regulated and only 11 up-regulated. A similar pattern was observed between 3 versus 10 dpi with 4307 down-regulated and 21 up-regulated genes and between 5 and 10 dpi where 1599 genes were down-regulated and 24 up-regulated (Fig. 2; Additional file 7).

### Over-represented GO terms in host DE gene clusters

**Fig. 3:**
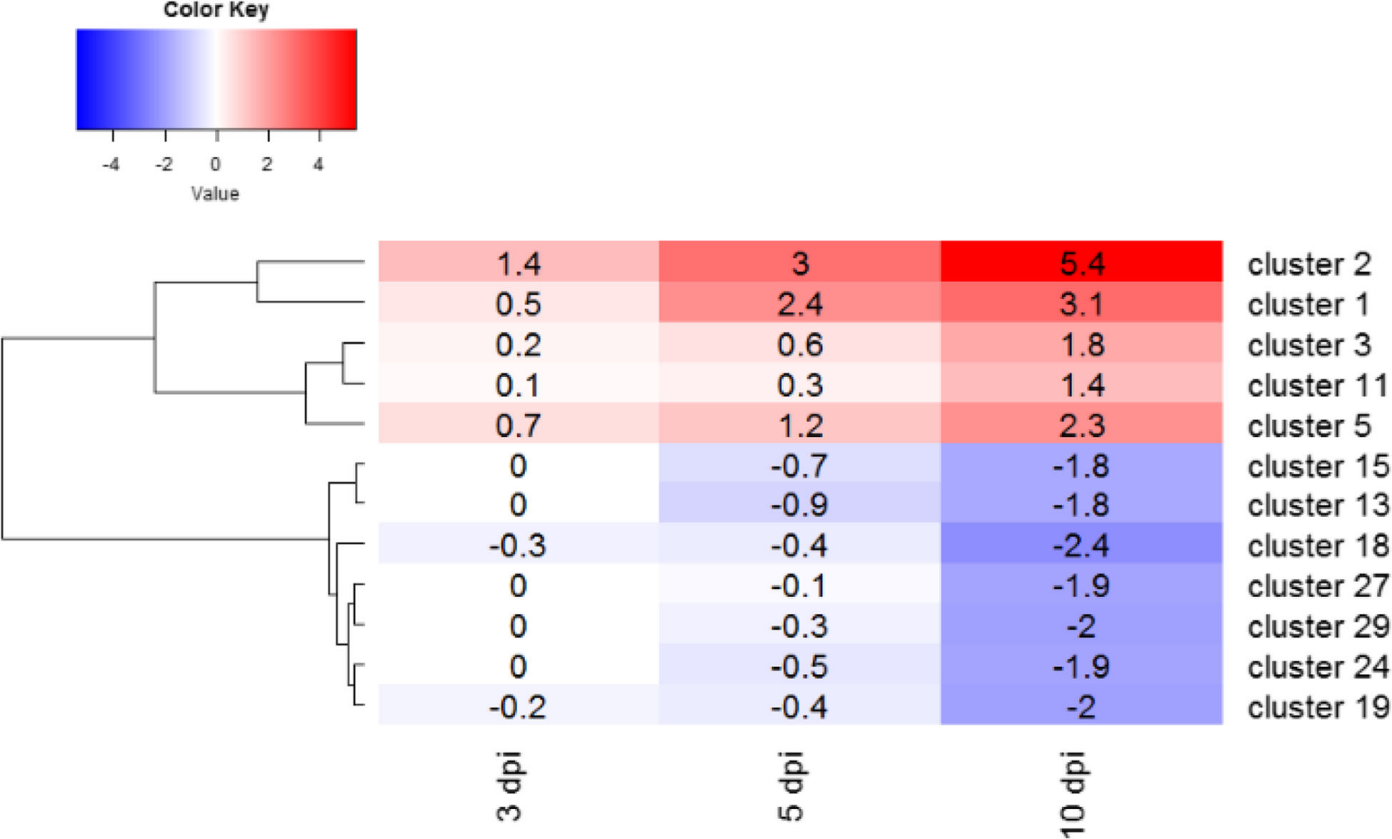
Heatmap representing clusters with gene ontology (GO) enrichment for the significant *Pinus pinaster* differential expressed (DE) genes (FDR < 0.05; |log_2_(Fold Change inoculated / mock-inoculated)| = 1) at each time point (3, 5 and 10 days post-inoculation)

Significant *Pinus pinaster* DE genes (13,323 genes) were classified in 53 clusters by using Hmisc R package. Due to the complexity, we set the |log_2_(Fold Change)| cut-off value to 1 (8802 DE genes) in order to reduce the number of clusters. DE genes were classified into 30 clusters, from which 12 had enriched GO terms and represent 92.7% of the DE genes (Fig. 3, Additional files 9 and 10).

Genes in cluster 1 were up-regulated at 5 and 10 dpi and slightly at 3 dpi. Terms included in the BP category were related to response to stimulus, response to chitin, regulation of reactive oxygen species (ROS), response to oxidative stress, positive regulation of cell death and phosphorylation transduction, all responses commonly related to biotic stress. Phytohormone signaling was also evident, since terms related to ET, JA and SA were detected in cluster 1, as well as systemic acquire resistance (SAR) mediated by SA. In the MF category, terms were related to glycosyltransferase activity, catalytic activity, lipase activity and lyase activity. In the CC category, only two terms were enriched, plasma membrane and cell periphery.

Genes in cluster 2 were highly up-regulated at all time points with an increase from 3 to 10 dpi, and highly up-regulated at 3 dpi in comparison with the other clusters. In the BP category we found terms related to cellular metabolic processes, cellular localization, cellular component organization or biogenesis, cytosolic transport, protein-containing complex subunit organization, vesicle-mediated transport and proteasome-mediated ubiquitin-dependent protein catabolic process. MF terms were mostly related to binding activities, protein binding, heterocyclic compound binding, nucleotide binding and phosphorylase and hydrolase activities. CC terms were related to protein containing complex, vacuole membrane, membrane coat, vacuole coat and site of polarized growth.

Terms in cluster 3 in the BP category were related to flavonoid and anthocyanin metabolic processes, hormone transport, response to oxidative stress, response to JA and auxin polar transport. No terms were enriched in the MF category. NADH dehydrogenase complex, vacuole and mitochondria were found in the CC category. Genes of this cluster were mostly up-regulated at 10 dpi.

In cluster 5 only 3 BP terms were enriched, all related to transport. In cluster 11, a response to ethanol term was overrepresented in the BP category.

Genes classified in clusters 18, 19, 27 and 29 were down-regulated mostly at 10 dpi and slightly at 3 and 5 dpi (Fig. 3). Terms in these clusters were related to growth, development, reproduction, morphogenesis and photosynthesis. Terms related to isoprenoid, terpenoid and carotenoid metabolic processes were found.

### Host phytohormone signaling

Four phytohormones seem to have a major role in *P. pinaster* defense response; they are JA, ET, SA and auxins. ABA, cytokinins (CK) and gibberellins seems to be suppressed. Log_2_(Fold Change) values for genes related to phytohormone signaling is provided as supplementary material (Additional file 11).

#### Jasmonic acid

**Fig. 4:**
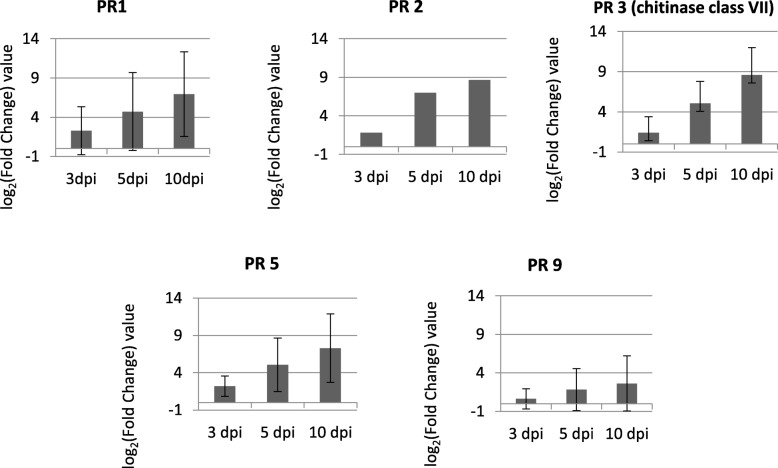
Expression pattern of differential expressed (DE) genes related to pathogenesis related (PR) proteins in *Pinus pinaster* inoculated seedlings. For each PR protein, the average of the log_2_(Fold Change inoculated/ mock-inoculated) value is represented at 3, 5 and days post-inoculation. Error bars represent the standard error of the mean

Lipoxygenases (*LOX*) and 12-oxo-PDA-reductase genes (*OPR*) were up-regulated at all time points in inoculated seedlings. An allene oxidase cyclase gene (*AOC*) was only up-regulated at 10 dpi, while allene oxidase synthase genes *(AOS*) were up-regulated at 5 and 10 dpi but also down-regulated at 10 dpi. A receptor Coronatine Insensitive 1 (*COI1*) gene was down-regulated at 5 and 10 dpi. A *JAZ* (jasmonate ZIM domain) gene, a repressor of JA signaling, was highly up-regulated at all time points. A *MYC* transcription factor gene, which negatively regulates expression of JA responsive genes, was down-regulated at 5 and 10 dpi. TOPLESS (*TPL*) and NOVEL INTERACTOR of JAZ (*NINJA*) corepressors were down- and up-regulated at 10 dpi, respectively. Jasmonate methyl transferase (*JMT*) genes were up-regulated at all time points, indicating JA conversion to methyl jasmonate (MeJA) for systemic signaling. JA induced genes include PR proteins with roles in fungal cell wall degradation, such as chitinases (PR3) and β-1,3-glucanases (PR2) [27]. A total of 15 chitinase genes, mainly belonging to chitinase class VII, and a *PR2* gene were up-regulated at all time points. Some peroxidases (PR9) can be induced by methyl jasmonate [26], and we detected up-regulation of 49 *PR9* genes at all times, especially at 10 dpi. However, 14 *PR9* genes were also down-regulated at 10 dpi (Fig. 4; Additional file 12).

#### Ethylene

In inoculated *P. pinaster* seedlings, 1-aminocyclepropane-1-carboxylic acid (ACC) synthase genes (*ACS*) were up-regulated at all time points while ACC-oxidase genes (*ACO*) were up-regulated at 5 and 10 dpi. The endoplasmic reticulum-associated receptor *ETR2* was up-regulated at 5 and 10 dpi; however, *ETR1* was down-regulated at 10 dpi. The downstream ETHYLENE INSENSITIVE 2 (*EIN2*), essential for positive regulation of ET signaling, was down-regulated at 5 and 10 dpi. *EIN3* and *EIL1* (*EIN3-LIKE1*), which act downstream of *EIN2*, were up-regulated at 5 and 10 dpi. *ERF/AP2* ethylene-responsive transcription factors were up-regulated at all time points, especially at 5 and 10 dpi. *EBF1* (EIN3-binding F-box protein 1), which degrades EIN3/EIL1 in the absence of ET, was down-regulated at 5 dpi.

#### Salicylic acid

Isochorismate synthase (ICS), the key enzyme involved in SA biosynthesis from the chorismate pathway, was down-regulated at 5 and 10 dpi. No differential expression of non-expressor of PR1 (*NPR1*) genes was found, however, genes of the TGA family transcription factors (*TGA*) were up-regulated at 10 dpi, suggesting SA signaling. SA can also be synthesized from the phenylalanine ammonia lyase (PAL) pathway, and *PAL* genes were up-regulated at all time points, although some *PAL* genes were also down-regulated. SA accumulation require two proteins, EDS1 (enhanced disease susceptibility 1) and PAD4 (Phytoalexin Deficient 4) [77]. Both *EDS1* and *PAD4* were up-regulated at 5 and 10 dpi. SA can be glycosylated by UDP-glycosyltransferase (UGT) to the inactive form 2–0-β-D-glucoside (SAG). Terms related to glycosyltransferase activity were enriched in cluster 1. PR1 is a PR protein commonly induced in defense response and its expression is SA responsive [107]. Three *PR1* genes were up-regulated at all time points (Fig. 4). PR5 is SA, JA and ABA-responsive [113] and 10 *PR5* genes were up-regulated at all time points, although two genes were also down-regulated (Fig. 4; Additional file 12).

#### Auxins

Two *YUCCA* (indole-3-pyruvate monooxygenase) genes were down-regulated at all time points, although one *YUCCA* gene was also up-regulated at 10 dpi. *TIR1* (Transport Inhibitor response), an auxin receptor [53], was up-regulated at 10 dpi. Two *Aux/IAA* (auxin/indole-3-acetic acid) family genes, which suppress the activity of transcriptional activators of the auxin response factor (ARF) family [99], were down-regulated at 10 dpi and 9 *ARF* genes were also down-regulated at 5 and 10 dpi. Small auxin up RNAs (*SAURs*), the largest family of auxin response genes, were up-regulated and down-regulated at 10 dpi, and several auxin-responsive genes were up- and down-regulated at all time points. *GH3* genes, which inactivate IAA, were both up- and down-regulated at 5 and 10 dpi. The enzyme IAA carboxyl methyltransferase (IAMT) catalyzes the conversion of IAA into the inactive form methyl IAA (MeIAA) [78] and four *IAMT1* genes were up-regulated at all dpi. Auxin efflux carriers PINFORMED (*PIN*) and P-glycoproteins (*PGP*) genes were down-regulated at 5 and 10 dpi, while an *AUX3* gene, an auxin influx transporter related gene, was up-regulated at all time points.

#### Abscisic acid

Some *9-cis-epoxicarotenoid dioxygenase* (*NCED*) genes, which participates in ABA biosynthesis, were up-regulated at all dpi, although one *NCED* gene was also down-regulated at 10 dpi. Zeaxanthin epoxidase (*ZEP*) was down-regulated at 10 dpi. Furthermore, ABA irreversible degradation was indicated since an *ABA 8-hydroxylase* gene was up-regulated at 5 and 10 dpi, which indicates ABA is converted to phaseic acid, with low activity. However, one *ABA 8-hydroxylase* gene was also down-regulated at 10 dpi. ABA signaling transduction is inhibited by type 2C protein phosphatases (PP2C), and *PP2C* genes were up-regulated at 5 and 10 dpi, although one gene was also down-regulated. Thus, ABA does not seem to have a major role in *P. pinaster* response to *F. circinatum*.

#### Gibberellic acid

Two key genes involved in GA biosynthesis, *ent-kaurene synthase* (*KS*) and *ent-kaurene oxidase* (*KO*), were down-regulated at 5 and 10 dpi. GA 20-oxidases (*GA20ox*) genes, involved in conversion of GA12 to the active forms of GA, was also down-regulated. GA 2-oxidases (GA2ox) convert bioactive gibberellins to their inactive form, and a *GA2ox* gene was highly up-regulated at all time points. These results suggest GA suppression.

#### Cytokinins

We found down-regulation of histidine kinase receptor (*HK*) genes at 5 and 10 dpi. HK ultimately activates a family of transcription factors, ARR, though no changes in *ARR* genes were detected. A *CK-O-glucosyltransferase* gene was up-regulated at 5 and 10 dpi, which participate in CK degradation. Therefore, CK signaling seems to be suppressed in inoculated seedlings at 5 and 10 dpi, with no CK signaling activity at 3 dpi.

#### Brassinosteroids

Some genes involved in BR biosynthesis were up-regulated at 5 and 10 dpi (*DET2*, *BR60X*) while others were down-regulated (*DWF4* and *DWF1*). A receptor-like kinase *BRI1* gene was down-regulated at 5 and 10 dpi. The downstream transcription factor *BES1/BZR1* was up-regulated at 5 and 10 dpi. Brassinosteroid-responsive RING-H2 (*BRH1*) was also up-regulated at all time points, however, BRH1 is not only involved in response to BR stimulus, but also in response to chitin, and we found up-regulation of *chitinase* at all dpi.

### Over-represented GO terms in pathogen DE gene clusters

**Fig. 5:**
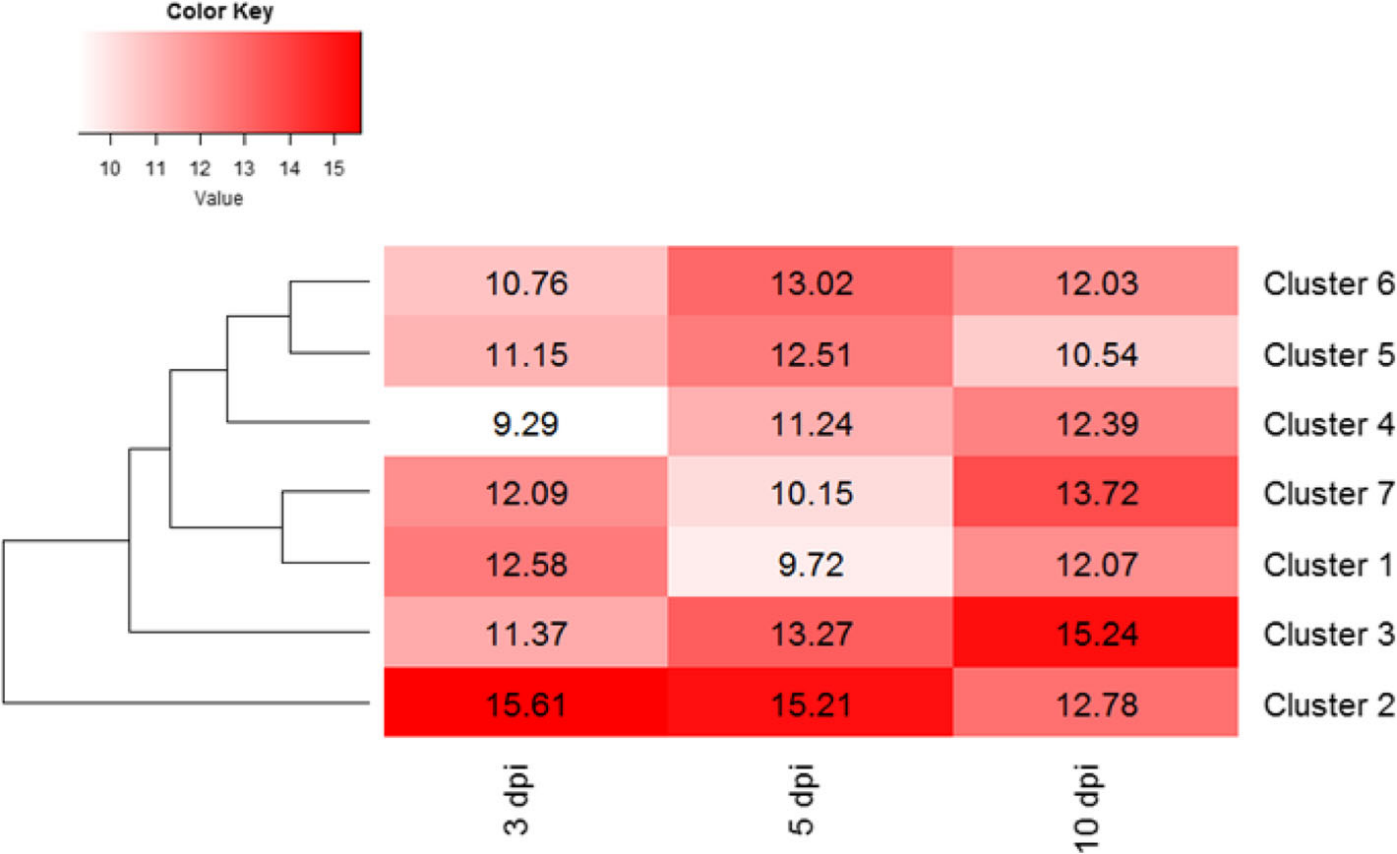
Heatmap representing clusters with gene ontology (GO) enrichment for *Fusarium circinatum* differentially expressed (DE) genes. Values are FPKM average at each time point (3, 5 and 10 days post-inoculation)

Significant DE genes for *F. circinatum* (FDR < 0.05; |log_2_(FoldChange)| > 0.5) were classified into 7 clusters (Fig. 5; Additional file 9) and 2 of them had enriched GO terms (Additional file 13). These two clusters (cluster 3 and 4) represent 98% of the DE gene dataset and almost all of them (92.07%) were classified into cluster 3. The pattern of expression of both clusters was similar, increasing from 3 to 10 dpi (Fig. 5).

In cluster 3, enriched BP terms were related to generation of precursor metabolites and energy, such as mitochondrial ATP synthesis coupled electron transport, establishment of protein localization, transport, intracellular transport, oxidative phosphorylation and oxidation-reduction process. In the CC category enriched terms were related to mitochondrion, ribosome, cytoplasm, organelle membrane and endoplasmic reticulum. Phospholipid binding, phosphatidylinositol binding, lipid binding and structural constituent of ribosome were enriched in the MF category.

In cluster 4 enriched terms were related to perception and metabolic processes. In the BP category, enriched terms were related to regulation of cellular and metabolic processes, regulation of cyclin-dependent protein serine/threonine kinase activity, positive regulation of biological processes and signal transduction. Site of polarized growth was the only term enriched in the CC category. Osmosensor activity was enriched in the MF category.

### Pathogen genes related to hormone production

**Fig. 6:**
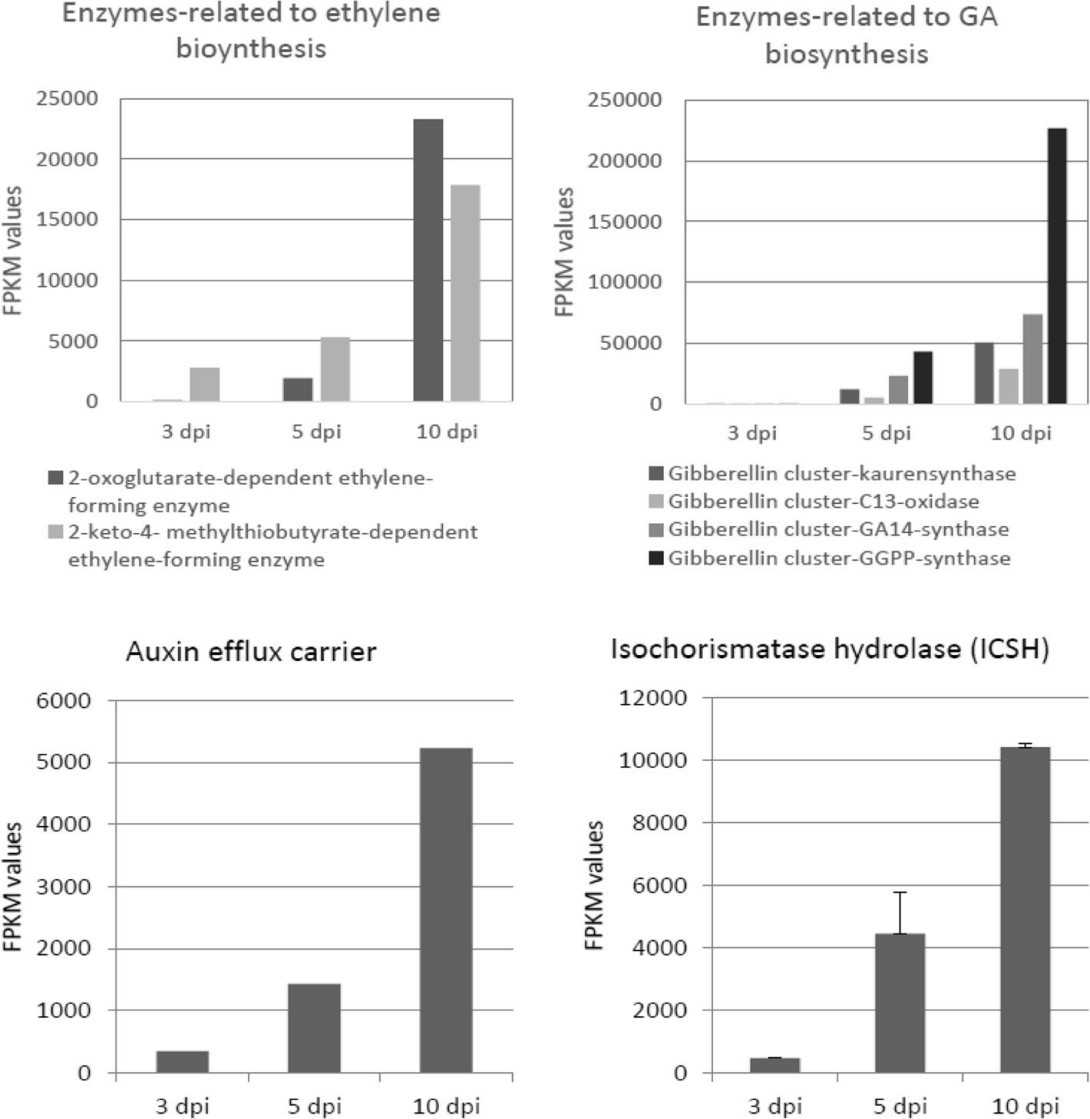
Expression profile of *Fusarium circinatum* genes related to virulence. For each gene, FPKM values at each time point (3, 5 and 10 days post-inoculation) are indicated

Two genes related to GA biosynthesis (*gibberellin cluster-C13-oxidase, gibberellin cluster-GA14-synthase*) were up-regulated by *F. circinatum* at all time points. Genes that participate in geranyl geranyl diphosphate (GGDP) and ent-kaurene synthesis (*gibberellin cluster-kaurensynthase and gibberellin cluster-GGPP-synthase*) were also up-regulated, with an increase from 3 to 10 dpi (Fig. 6; Additional file 14).

Two genes (*2-oxoglutarate-dependent ethylene succinate-forming enzyme* and *2-keto-4-methylyhiobutyrate-dependent ethylene-forming enzyme*) coding enzymes involved in fungal ET biosynthesis were up-regulated by *F. circinatum* at all time points, especially at the latter stage (Fig. 6). An auxin efflux gene was also up-regulated at all time points (Fig. 6, Additional file 14).

Isochorismatase hydrolase (ICSH) family proteins catalyze the hydrolysis of isochorismatase, a key metabolite for SA biosynthesis from the chorismate pathway. Two *ICSH* genes were up-regulated by *F. circinatum* and showed increased expression over time (Fig. 6, Additional file 14).

From the DE *F. circinatum* genes mentioned above, 4 of them showed hits to the Pathogen Host Interaction (PHI) database with E-value <1e^− 4^ (Additional file 15). Knockout of these genes in other pathogens resulted in reduced virulence in their hosts. Interestingly, one of these genes (*2-keto-4-methylyhiobutyrate-dependent ethylene-forming enzyme*) showed 90% similarity to a *F. oxysporum* gene, which when knocked out resulted in reduced virulence in tomato plants [74].

## Discussion

In forestry, sequencing of genomes is still a challenge, particularly for pines due to large genome sizes, retrotransposons and repeats. Therefore, transcriptome sequencing has become a good alternative for generating genomic resources, especially useful in understanding host-pathogen interactions [1, 114, 115]. In the present work, we generated a high quality de novo *Pinus pinaster* reference transcriptome constituted by 24,375 sequences, from which 17,523 were full length genes. Comparison of BUSCO results of the *P. pinaster* transcriptome with published *Pinus* transcriptomes showed high completeness and contiguity of the assembly [108, 110]. In total, 73% of reads mapped to the host transcriptome while 2% mapped to the pathogen, similar to results obtained in other studies [66]. Therefore, this high-quality shoot transcriptome represents a valuable resource for further research on maritime pine trees.

In addition, the present study provides a comprehensive characterization of the underlying molecular mechanism for defense and pathogenicity response in the *Pinus pinaster* - *Fusarium circinatum* pathosystem. The moderate resistance maritime pine has shown to the pathogen [49] can be explained, at least in part, by the early induction of defense-related genes and complex phytohormone signaling including SA, JA and ET. We also hypothesized key steps where the pathogen could be manipulating host defenses to its own benefit by altering host hormone homeostasis.

The early recognition and activation of *P. pinaster* defense responses can be deduced from genes classified in cluster 2, up-regulated at 3 dpi, before the fungus has penetrated within the host tissue. At 5 and 10 dpi, when the fungus has penetrated and invaded the host tissue, the expression of genes in cluster 1 highly increased and enriched GO terms related to phytohormone signaling, regulation of ROS, oxidative stress, positive regulation of cell death and signal transduction were found. This indicates activation of classical pattern triggered immunity (PTI) in response to the pathogen [11]. Terms related to chitinase activity were over-represented in this cluster and chitinases are commonly induced by pathogen attack in several trees such as *P. abies*, *Pinus elliottii*, *Pinus sylvestris* and *Fagus sylvatica* [27, 47, 71, 86, 88]. Rice and *Arabidopsis* perceive fungal chitin through the lysine motif (LysM) RLK CERK1 which induces CERK1 dimerization, essential for the activation of downstream signaling [67, 112]. We found up-regulation of genes encoding LysM motif RLK and chitinase in inoculated seedlings at 3 dpi, suggesting fungal recognition at the early stage. Furthermore, oligosaccharides released from chitin degradation can serve as pathogen-associated molecular patterns (PAMPs) for activation of PTI signaling in the host [68, 81].

Several studies have demonstrated the crucial role of phytohormones in host defense response [77]. In *P. pinaster* - *F. circinatum* pathosystem terms related to ET, JA and SA were over-represented in cluster 1. By contrast, GA, CK and ABA signaling seemed to be suppressed. Genes involved in JA and ET biosynthesis were up-regulated from 3 dpi and both phytohormones have been shown to cooperate under biotic stress conditions [9, 61, 62]. Despite the active biosynthesis of JA, the down-regulation of *COI1* and *MYC2* at 5 and 10 dpi, together with the up-regulation of *JAZ* genes reflect a block of JA signaling. COI1 has an F-box domain and is involved in the formation of Ubiquitin ligase E3 SCF complex, for protein degradation [30]. In the presence of JA-Ile, COI1 degrades JAZ proteins, which are direct targets of the SCF COI1 E3 ubiquitin-ligase [22, 98]. In the absence of COI1, JAZ proteins repress MYC transcription factor suppressing the expression of JA responsive genes. Based on the fact that COI1 is a key element for regulation of the JA signaling pathway [30] and the importance this phytohormone has shown in defense response against several necrotrophic pathogens [41], we hypothesize that *F. circinatum* could be blocking JA signaling by *COI1* suppression. Similarly, Thatcher et al. [96] suggested that *F. oxysporum* hijacks COI1-mediated JA signaling to promote disease in *Arabidopsis* plants.

The induction of genes related to ET biosynthesis, together with the up-regulation of ethylene-responsive regulator genes *ERF*, *ETR* and *EIN3/EIL1* suggests an active role of this phytohormone in *P. pinaster* defense response. However, the down-regulation of *EIN2* at 5 and 10 dpi, essential for positive regulation of ET, could interfere with ethylene signaling. Interestingly, we found up-regulation of 2 genes related to ET biosynthesis in *F. circinatum* which could have a role in perturbing ET homeostasis in the host. Similarly, De Vleesschauwer et al. [28] reported that ET produced by the rice brown spot pathogen *Cochliobolus miyabeanus* is a virulence factor and suggested that fungal ET interferes with rice ET signaling to suppress effective defense pathways. Indeed, one of the two genes related to ET production in *F. circinatum* showed hits to the PHI database and knockout of this gene in *F. oxysporum* resulted in a reduced virulence phenotype in common bean plants [74].

ET and JA participate in induction of certain PR proteins [6, 26, 113]. In the present study, we detected up-regulation of *PR1*, *PR2*, *PR3*, *PR5* and *PR9* genes. Rakwal et al. [79] reported a coordinated increase in ET and some PR3 in rice plants. Similarly, Carrasco et al. [18] found synchronized increase between the induction of *PR5* and ET in *P. radiata* seedlings inoculated with *F. circinatum*. PR2, PR3 and PR5 participate in degradation of glucans of the cell wall of the pathogen, making the fungus more susceptible to cell lysis and attack by other plant defense molecules [46, 107]. Similarly, during *Fusarium culmorum* infection, two basic isoforms of PR2 and three basic isoforms of PR3 were induced in germinating wheat seeds upon infection [19]. An increase of a class III PR9 responsible for lignin biosynthesis and cell wall thickening was detected in *Pinus sylvestris* roots infected with *Heterobasidion annosum* [1]. Indeed, induction of PR5 in *P. radiata*, highly susceptible to the pathogen, infected by *F. circinatum*, occurs after 6 dpi, but no differences were found at 3 dpi between inoculated and mock-inoculated samples [18]. Here we detected up-regulation since 3 dpi, before the pathogen has penetrated, suggesting quick activation of defense responses in *P. pinaster* against *F. circinatum*.

SA biosynthesis seems to occur from the PAL pathway and not from the chorismate pathway, since *ICS* was down-regulated at 10 dpi and not differentially expressed at 3 and 5 dpi. Similarly, Ding et al. [31] found induction of *PAL* in a wheat variety after *Fusarium graminearum* infection, while *ICS* was down-regulated, suggesting SA accumulation via the phenylpropanoid pathway. Interestingly, we found up-regulation of two *ICSH* genes by *F. circinatum*, which catalyzes the hydrolysis of ICS. Moreover, ICSH1 has been shown to accumulate in a highly aggressive *Verticillium dahliae* isolate but not in a weakly aggressive isolate [34]. Indeed, cotton plants inoculated with a *V. dahliae* mutant lacking the *ICSH1* gene resulted in attenuated aggressiveness while higher levels of SA were detected [57]. Zhu et al. [123] propose that ICSH1 is a virulence factor in *V. dahliae* and that the high level of ICSH in highly aggressive isolates represses the SA pathway in potato plants by hydrolyzing ICS. Similarly, we suggest that *F. circinatum* prevents SA biosynthesis from the chorismate pathway, although whether ICSH is a virulence factor in *F. circinatum* requires further investigation. In addition, *TGA*, *EDS1* and *PAD4* were up-regulated in inoculated *P. pinaster* seedlings, suggesting SA accumulation has a role in the host defense response. Furthermore, PAD4 is also required for production of the phytoalexin camalexin and PR1 synthesis. *PR1* gene expression is SA responsive and three *PR1* genes were up-regulated at all dpi. Carrasco et al. [18] also found *PR1* genes highly up-regulated in *F. circinatum* resistant *P. radiata* genotypes.

Van der Does et al. [106] proposed that suppression of the JA pathway by SA functions downstream of the E3 ubiquitin-ligase Skip-Cullin-F-box complex SCF^COI1^. However, cooperation between JA and SA has been also reported [36, 95]. Due to the up-regulation of genes related to SA and JA biosynthesis in inoculated *P. pinaster* seedlings and based on the fundamental role JA has shown against necrotrophic pathogens [41], we suggest cooperation of SA and JA rather than an antagonism in the *P. pinaster*-*F. circinatum* interaction.

Genes encoding enzymes that catalyze the methylation of JA (MeJA) were up-regulated at all time points suggesting a role of systemic signaling in *P. pinaster* defense response. Furthermore, “SAR mediated by SA” term was over-represented in cluster 1. Truman et al. [102] suggested that JA has an earlier role in SAR establishment, before SA accumulation in *Arabidopsis.* Similarly, we found up-regulation of genes related to JA biosynthesis (*LOX, OPR*) since 3 dpi, before induction of SA related genes, mostly up-regulated at 5 and 10 dpi (*NPR1*, *TAG*, *PAD4*, *EDS1*), suggesting again, possible cooperation between the two phytohormones. Foliar application of MeJA in *P. radiata* conferred increased resistance to the necrotroph *Diplodia pinea* [42] and against other pathogenic fungi in *P. abies* [39, 54]. However, MeJA pre-treatment was ineffective in protection against *F. circinatum* in *Pinus patula* [37] and *P. pinaster* seedlings [111]. Nevertheless, the high induction of MeJA producing genes in *P. pinaster* inoculated seedlings indicates a role in defense response against the pathogen. Indeed, Sasaki et al. [85] described MeJA as a key component in the jasmonate signaling pathway by controlling its own expression via a feedback mechanism by inducing the expression of LOX and AOS, both enzymes that catalyze key steps in JA biosynthesis. This can explain the up-regulation of JA biosynthesis genes in spite of the suppression of JA signaling. Furthermore, studies have reported the role of MeJA in chalcone synthase (CHS) induction in soybean and parsley [25] and *Picea glauca* [82]. CHS is a key enzyme in the flavonoid biosynthesis pathway and flavonoids play an important role in plant defense against pathogens. We found up-regulation of *CHS* at all dpi in *P. pinaster* inoculated seedlings, with an increase from 3 to 10 dpi.

Although SA, JA and ET are key players in *P. pinaster* response to *F. circinatum*, auxins can also have a role since some *SAUR* genes and a *TIR1* gene were up-regulated. However, we also detected suppression of auxin biosynthesis and signaling (down-regulation of *indole-3-pyruvate monooxygenase* and *ARF*), as well as accumulation and conjugation to inactive forms inside the cell (up-regulation of *GH3* and a *AUX* gene encoding for influx proteins, and down-regulation of *PIN* and *PGP* genes encoding efflux proteins). Nevertheless, *IAMT1* genes were up-regulated suggesting conversion of IAA into the inactive MeIAA form. Crosstalk between auxins and other phytohormones has been documented and an inhibitory effect of auxins on JA signaling in *Arabidopsis* seedlings has been reported [56]. Moreover, auxins are inducers of expansins, involved in cell wall extensibility [20] which could enhance pathogen penetration. Indeed, auxins mostly are related to growth and development which are functions sacrificed by the plant to favor mechanisms involved in defense responses. This trade-off between growth and defense is reflected by over-represented GO terms related to growth, morphogenesis and photosynthesis for clusters with down-regulated DE genes (clusters 18, 19, 27 and 29; Fig. 3). Nonetheless, the role of auxins in *P. pinaster* defense response to *F. circinatum* is not clear and needs to be elucidated.

Furthermore, a gene related to auxin efflux was up-regulated by *F. circinatum* at all time points, suggesting auxin accumulation could have a role in virulence. Similarly, an over-accumulation of IAA in *F. oxysporum* was related to a hypervirulent phenotype on Orobanche [24]. The two-fold reduction of a gene required for auxin biosynthesis in *Puccinia graminis* f. sp. *tritici* led to a decrease in pustule formation [119]. GA production is common in species of the FFC. Although Malonek et al., [64] reported that only one gene in the GA biosynthetic cluster is present in *F. circinatum*, we found two potential genes, related to GA biosynthesis, up-regulated. These two genes also had hits to the PHI database, and knockout of these genes in other pathogens resulted in reduced virulence and loss of pathogenicity phenotypes in the host. However, these results must be considered with caution, since percentages of identity ranged from 22 to 29%.

By 10 days after inoculation, when the fungus has invaded the host tissue and the lesion in the shoot tip is evident, an additional defense response seems to occur in *P. pinaster* seedlings (cluster 3). GO terms related to flavonoids and anthocyanins biosynthesis, secondary metabolites involved in defense responses, were over-represented [7, 100]. Despite the effort of the plant in synthesizing these secondary metabolites at the latter stage of infection, the host is not able to counteract fungal infection.

This study highlights the importance of phytohormones in the *P. pinaster-F. circinatum* interaction. One possibility would be to use hormone application to induce resistance. While previous results show the application of MeJA at various concentrations does not affect lesion development of *F. circinatum* inoculated *P. pinaster* seedlings [111], this study points to SA in combination with MeJA as a potential strategy to investigate.

## Conclusions

This work provides knowledge of mechanisms underlying the *P. pinaster* defense response against *F. circinatum,* indicating activation of defense mechanisms from as early as 3 dpi, by induction of PR genes and mainly regulated by a complex signaling pathway involving crosstalk between SA, JA and ET. Moreover, we hypothesize key steps where the pathogen could be manipulating host defense in its favor, mainly by perturbing phytohormone signaling homeostasis in the host. Future work in measuring SA, JA, ET and auxin content *in planta*, as well as functional studies with *F. circinatum* mutants, will be necessary to support this hypothesis.

## Methods

### Plant and fungal material

Six-month-old *P. pinaster* seedlings purchased at ‘Eskalmendi’ nursery (Alava, Spain) were used for the experiment. These seedlings were grown in this nursery from seeds of *P. pinaster*, provenance Landas. Seedlings were maintained in a greenhouse at 20–22 °C with a photoperiod of 12 h light / 12 h darkness and inoculated after 2 weeks of acclimation.

For inoculations, a virulent *F. circinatum* isolate from Basque Country (Northern Spain) (Isolate CECT20759, isolated from a *P. radiata* tree) [50] was used. A fungal spore suspension in sterile distilled water was prepared after 1 week of culture on Potato Dextrose Agar (PDA) by scraping the plate surface and passing through two layers of glass wool. Spore concentration was measured with a hemocytometer and adjusted to 5 × 10^5^ spores/ml.

### Inoculation and microscopic observation

Ninety six-month-old *P. pinaster* seedlings were inoculated with a green fluorescent protein (GFP)-tagged strain of *F. circinatum*. Agrobacterium-mediated transformation of isolate CECT20759-GFP was performed as previously described [69, 80] using the pRF-gGFP plasmid [75]. This plasmid contained the sGFP coding sequence under the control of the *A. nidulans* Pgpd promoter excised from plasmid gGFP [65]. The first 2 cm of the shoot tip were excised and a 2 μl drop of the spore suspension (1000 conidia) was deposited in the wound with a micropipette. A set of 54 seedlings were mock-inoculated with sterile distilled water. Plants were covered with a plastic bag for 24 h in order to maintain high humidity and favor fungal infection. During the first 8 days following inoculation, as well as at 10, 14 and 17 dpi, plant tissue from 6 inoculated and 2 mock-inoculated plants per day were visualized under an epifluorescence microscope (Nikon EFD-3). Cross-sections were placed on glass slides, covered with cover glasses and visualized under the epifluorescence microscope. Sections were taken from the point of inoculation and progressively downward until no pathogen was visualized, a minimum of four sections were visualized for each plant. Progression of pathogen growth within host tissue served to determine the times of sampling for RNAseq analyses.

### Inoculation and tissue sampling for RNA extraction

A total of 288 *P. pinaster* seedlings were used for the experiment. Half of them were inoculated with the pathogen as explained above, while the remaining half were mock-inoculated with sterile distilled water. For sampling, the top 1.5 cm of shoot tissue was harvested for each seedling at three different times: 3, 5, and 10 dpi, for both inoculated and mock-inoculated seedlings. We used 4 biological replicates (BR) per group (mock-inoculated and inoculated) with a pool of 8 individuals each. Plant material was immediately frozen in liquid nitrogen and stored at − 80 °C until use.

A total of 16 inoculated and 16 non-inoculated seedlings were maintained for visualizing disease progress. Lesion length was measured at the end of the experiment (33 dpi). Additionally, 3 cm of the tip of 6 randomly collected plants of each group (inoculated and mock-inoculated) were surface sterilized by immersion in 70% EtOH for 1 min. Plant tissue was transversally cut and cultured on Fusarium Selective Medium [2] to verify efficacy of the inoculation.

### RNA isolation and sequencing

Total RNA was extracted using a Plant/Fungi Total RNA Purification Kit (Norgen Biotek Corp., Thorold, Ontario) following the manufacturer’s instructions and stored at − 80 °C. The protocol included a DNase treatment step for removal of residual DNA (Norgen’s RNase-free DNase I Kit). The integrity of extracted RNA was assessed using a 2100 Bioanalyzer (Agilent Technologies). RNA with an RNA Integrity Number (RIN) > 7 was considered good quality. Two biological replicates for inoculated samples at 10 dpi did not pass the RIN threshold and were excluded for the rest of the analysis, likely due to necrosis of the tissue (Additional file 16).

Approximately 40 μl of total RNA for each sample was submitted to Macrogen (Macrogen Korea). Twenty-four TruSeq mRNA stranded libraries with polyA selection and an insert size of 300 bp was prepared. One hundred one bp paired-end reads were generated with Illumina HiSeq 4000, with a sequencing depth of 81–181 million reads per sample. Samples were multiplexed in 4 lanes, with 6 samples per lane. In order to avoid technical errors due to sample position in the sequencer, each biological replicate of each sample was included in a different lane.

### Raw data quality control and filtering

Quality control of raw Ilumina reads was performed by FastQC analyses v0.11.7 [3]. Trimmomatic 0.36 was used for trimming and filtering of low-quality reads and adapter removal [10]. Bases with a Phred Score below 30 and reads shorter than 40 bp were removed. The first 15 bases of all reads were trimmed to remove sequencing biases. The quality of trimmed and filtered reads was checked again by FastQC (Additional file 16).

### Reference transcriptomes

#### *Pinus pinaster* de novo transcriptome assembly

##### Preliminary assemblies

Diverse studies have shown a high variability in the assembly of reads when using different assemblers [13, 122]. Therefore, the de novo transcriptome assembly of *P. pinaster* was created using two different assemblers: Trinity v.2.4.0 [43] and transABySS v.2.0.1 [83], programs specially developed for de novo transcriptome assembly of RNAseq short-read data. A multi kmer strategy was adopted; De Bruijn graphs were built over different kmer values and the resulting assemblies merged to improve sensitivity and accuracy of gene set reconstruction [33, 93, 122].

Seven assemblies were created with Trinity with kmer values of 19, 21, 23, 25, 27, 29 and 31 and the next set of parameters: minimum contig length of 350 bp and maximum read coverage of 50 for in silico normalization. A further 5 assemblies were generated with kmer sizes of 19, 21, 23, 25 and 27 without normalization of the reads. For TransABySS, we ran 9 different de novo assemblies with a kmer value ranging from 21 to 77, with a step size of 8, as well as a kmer value of 25. The minimum output sequence length was set to 350 bp for all TransABySS assemblies. Quality of each preliminary assembly was checked using Transrate v.1.0.3 [91].

##### Merging assemblies

Best quality preliminary assemblies were merged into one dataset using the EvidentialGene tr2aacds pipeline version 2017.12.21 [40], which builds the optimal assembly from a pool of different assemblies. It uses fastanrdb of exonerate package v 2.2.0 [90] for pairwise sequence comparison based on protein qualities for predicting the best coding DNA sequences (CDS) among identical sequences and reduce redundancy. Then, cd-hit-est v.4.7 [55] and BLASTn (blast v.2.7.0) [121] are used to cluster nucleotide sequences with 98% similarity into loci. EvidentialGene tr2aacds returns the subset of most accurate coding genes, classified into alternate or main (pimary) CDS. Alternate CDS for each gene were discarded. Additionally, an “okay” or “drop” value is assigned using scores of alignment and protein quality to separate “useful” and “not useful” transcripts, respectively. The “drop” class contains redundant and uninformative mRNA transcripts and they were discarded for the rest of the analyses. The quality of the transcript sequences derived from the merged assembly were checked by Transrate v.1.0.3.

##### Annotation

Both reference transcriptomes were annotated using the Eukaryotic Non-Model Transcriptome Annotation Pipeline (EnTap) version 0.8.2 [44]. Coding regions of the transcripts were selected by GenemarkS-T v5.1 March 2014 [94]. The annotation process integrates similarity search across different databases with a minimum query and target coverage of 80 and 60%, respectively. NCBI non-redundant protein (release 2018–03), RefSeq (release 87), SwissProt (release 2018–03) and Arabidopsis proteome (release 2018.03) databases were used for BLASTp alignment using Diamond 0.9.9 [16]. Non-pine origin sequences were removed from the assembly by significant alignment to fungal, bacterial, viral, insect, archaea, opistokhonta and amoebozoa sequences. For orthologous group and gene ontology (GO) assignment InterProScan v5.28–67.0 [51] and EggNOG v0.12.7 [48] were used. Finally, all contaminants and non-frame selected and unannotated sequences were manually filtered. Kyoto Encyclopaedia of Genes and Genomes (KEGG) orthology (KO) annotation was also assigned by using GhostKOALA [52].

Mercator [60], with default parameters and including all the databases available, was used to assign predicted proteins into MapMan v.3.5.1R2 [97] bins, a tool that allows the visualization of metabolic pathways and processes of a large set of data.

##### Assembly validation

Completeness and contiguity of the assembly was checked using BUSCO v3.0.2 (Benchmarking of Single-Copy Orthologs) [89]. We used the eukaryote_odb9 and embryophyta_odb9 lineages to identify putative universal single copy orthologs (USCOs) in the assembly.

#### *Fusarium circinatum* reference transcriptome

The *F. circinatum* reference transcriptome was obtained from the *F. circinatum* (strain FSP34) genome sequence (B. D [117].) by extracting the longest transcript sequence for all predicted genes (15,049). A total of 14,185 sequences were annotated with EnTAP, of which 5368 were assigned GO terms [109]

### Mapping, differential expression (DE) and gene ontology (GO) enrichment analysis

The *F. circinatum* transcriptome and *P. pinaster* de novo transcriptome assembly were combined in a single dataset to account for cross species mapping [72]. This dataset was used to map reads with Kallisto v.0.44.0 [15] with sequence bias correction and bootstrap samples set to 100. Kallisto abundance output files (transcript abundance estimates) for each read was imported to R 3.5.1 with tximport v.1.6.0 [92]. DESeq2 v.1.18.1 [63] was used for DE analysis, with a FDR < 0.05 and |log2(Fold change)| > 0.5. Transcripts with less than 20 reads in at least 3 samples were filtered for the rest of the analysis. For host DE analysis *F. circinatum* data was removed and expression levels at different time points (3, 5 and 10 dpi) were compared between inoculated and mock-inoculated samples. For *F. circinatum* DE analyses, *P. pinaster* data was filtered and gene expression levels between time points were compared between the inoculated samples (3 versus 5 dpi, 3 versus 10 dpi and 5 versus 10 dpi). To excluded potential endophyte contamination and confirm the confidence of *F. circinatum* expressed genes, a high confidence DE analysis (inoculated relative to mock-inoculated) with all the data (host and pathogen) was performed and then compared to the one with only inoculated samples. Genes considered not high confident at all time points were discarded. GOSeq v.1.34.0 [120] was used to identify GO terms significantly overrepresented (FDR < 0.10) in the DE data. GO enrichment was based on the annotated transcriptomes for each species.

PCA was performed for *P. pinaster* and *F. circinatum* normalized read counts (regularized-logarithm transformation or rlog - DESeq2 rlog function) for all samples, to visualize the overall effect of experimental covariates and batch effects.

Significant DE genes for each species were clustered based on the FPKM (Fragments Per Kilobase of transcript per Million mapped reads) values using Hmisc v.4.1–1, with a Pearson correlation of 0.5. Overrepresented GO terms for each cluster were identified with GOSeq. *F. circinatum* genes were subjected to a PHI-BLAST analysis using the PHI database 4.2 [104] to identify potential pathogenicity and virulence factors. Default parameters were employed and lowest e-value hits were considered further.

## Supplementary information

**Additional file 1 **Symptoms at the shoot tip of inoculated (left side) and mock-inoculated (right side) *Pinus pinaster* seedlings by the end of the experiment (33 dpi).

**Additional file 2.** Statistics for each TransABySS and Trinity assembly. N seq: number of transcripts; N bases: number of bases; Mean length: mean length of the transcripts; N50: N50 value; Ns: number of unknown bases; % GC: guanine and cytosine content; trinity-N: in silico normalized trinity assembly; trinity-nN: non-normalized trinity assemblies. * Best quality preliminary assemblies selected to generate the final assembly.

**Additional file 3.** Comparative statistics between normalized (Norm) and non-normalized (N-norm) Trinity preliminary assemblies. Kmer value; % of mapped fragments; % of good mapping; AS: assembly score; OP: optimal score; OC: optimal cutoff; Number of good contigs; % good contigs.

**Additional file 4. **BUSCO analysis against the embryophyta lineage database comparing the last *Pinus* de novo transcriptomes published*. P. patula* v1.0 [110]; *P. patula* v2.0 and *P. tecunumanii* [108].

**Additional file 5. ***Pinus pinaster* de novo transcriptome annotation.

**Additional file 6. ***Pinus pinaster* de novo transcriptome annotation by Mercator tool.

**Additional file 7. **mapped reads for each species. Number of differential expressed (DE) genes for *Pinus pinaster* and DE genes for *Fusarium circinatum* at each time point in inoculated samples (FDR < 0.05; |log2(Fold Change)| > 0.5). Ppin: *P. pinaster*; Fcir: *F. circinatum*;HC: high confident.

**Additional file 8. **Principal component analyses (PCA) for *Pinus pinaster* (above) and *Fusarium circinatum* (below) rlog data of the differential expression gene analysis (DESeq2). In red: mock-inoculated samples; in blue: inoculated samples at 3 dpi; in green: inoculated samples at 5 dpi; in yellow: inoculated samples at 10 dpi.

**Additional file 9. **Clustering of *Pinus pinaster* and *Fusarium circinatum* differential expressed (DE) genes. For each cluster with gene ontology (GO) enriched terms, number of genes and percentage for genes are indicated.

**Additional file 10. **Significantly enriched GO terms identified from *Pinus pinaster* genes in each cluster.

**Additional file 11: **Phytohormone related differentially expressed (DE) genes in *Pinus pinaster*.

**Additional file 12: **Pathogenesis related (PR) genes differentially expressed (DE) in *Pinus pinaster*.

**Additional file 13: **Significantly enriched GO terms identified from high confidence expressed *Fusarium circinatum* genes.

**Additional file 14: **Hormone related differential expressed (DE) genes in *Fusarium circinatum*.

**Additional file 15: ***Fusarium circinatum* DE genes related to hormone production with hits in the Pathogen Host Interaction (PHI) database.

**Additional file 16:.** RNA-seq data statistics for each sample at each time point, before and after filtering and trimming. Dpi: days post-inoculation; BR: biological replicate, RIN: RNA Integrity Number; Q 30: Phred quality score 30.

## Data Availability

The datasets generated and analysed during the current study are available in the Sequence Read Archive (SRA) repository, accessible through BioProject accession PRJNA543723.

## Abbreviations

ABA: Abscisic acid
ACC: 1-aminocyclepropane-1-carboxylic acid
ACO: ACC-oxidase
ACS: ACC synthase
AOC: Allene oxidase cyclase
AOS: Allene oxidase synthase
ARF: Auxin response factor
Aux/IAA: Auxin/indole-3-acetic acid
BP: Biological process
BR: Biological replicate
CC: Cellular compartment
CHS: Chalcone synthase
CK: Cytokinins
COI1: Coronatine Insensitive 1
DE: Differentially expressed
Dpi: Days post-inoculation
EBF1: EIN3-binding F-box protein 1
EDS1: Enhanced disease susceptibility 1
EIN2: Ethylene insensitive 2
ET: Ethylene
ETR: Endoplasmic reticulum-associated receptors
FPKM: Fragments Per Kilobase of transcript per Million mapped reads
GA: Gibberellic acid
GA20ox: GA 20-oxidases
GA2ox: GA 2-oxidases
GFP: Green fluorescent protein
GGDP: Geranyl geranyl diphosphate
GH3: Gretchen Hagen 3
GO: Gene ontology
HC: High confident
HK: Histidine kinase
IAA: Indol-3-acetic acid
IAM: Indol-3-acetamide
IAMT: IAA carboxyl methyltransferase
ICS: Isochorismate synthase
ICSH: Isochorismatase hydrolase
JA: Jasmonic acid
JA-Ile: Jasmonoyl-isoleucine
JAZ: Jasmonate ZIM domain
JMT: Jasmonate methyl transferase
KO: Ent-kaurene oxidase
KS: Ent-kaurene synthase
LOX: Lipoxygenase
MeIAA: Methyl IAA
MeJA: Methyl jasmonate
MF: Molecular function
NCED: 9-cis-epoxicarotenoid dioxygenase
NINJA: Novel interactor of JAZ
NPR1: Non-expressor of PR1
OPR: 12-oxo-PDA-reductase
PAD4: Phytoalexin deficient 4
PAL: phenylalanine ammonia lyase
PAMPs: Pathogen-associated molecular patterns
PCA: Principal component analysis
PFP: Phosphorylase family protein
PGP: P-glycoproteins
PHI: pathogen-host interactions
PIN: Pinformed
PP2C: 2C protein phosphatases
PR: Pathogenesis-related
PTI: Pattern triggered immunity
RIN: RNA Integrity Number
ROS: Reactive oxygen species
SA: Salicylic acid
SAG: 2–0-β-D-glucoside
SAM: S-adenosyl-L-methionine
SAR: Systemic acquire resistance
SAURs: Small auxin up RNAs
TIR1: Transport Inhibitor response
TPL: TOPLESS
UGT: UDP-glycosyltransferase
ZEP: Zeaxanthin epoxidase
