# Supplementary material for: The transcriptome of Pinus pinaster under Fusarium circinatum challenge
Source: BMC Genomics. 2020 Jan 8;21:28. doi: 10.1186/s12864-019-6444-0 (PMC6950806; doi:10.1186/s12864-019-6444-0)
Supplement: Supplementary file 1 — Additional file 1 Symptoms at the shoot tip of inoculated (left side) and mock-inoculated (right side) Pinus pinaster seedlings by the end of the experiment (33 dpi). [file 12864_2019_6444_MOESM1_ESM.pdf]

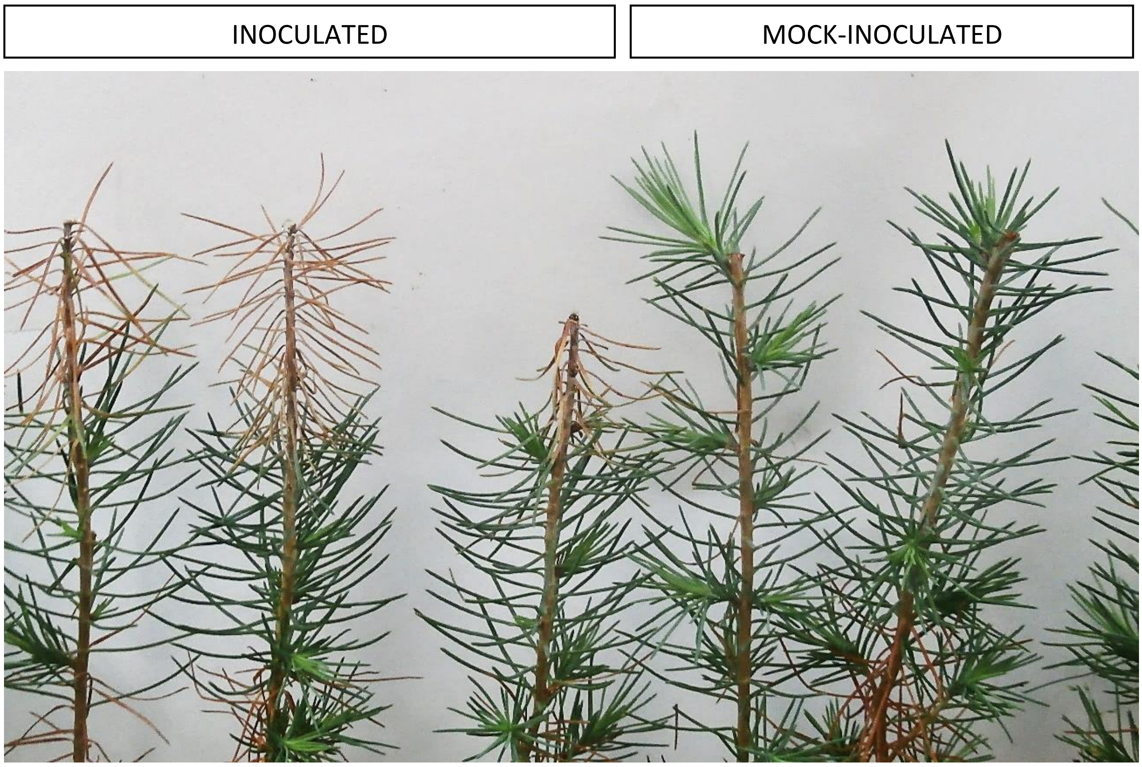

Additional file 1: Symptoms at the shoot tip of inoculated (left side) and mock-inoculated (right side) *Pinus pinaster* seedlings by the end of the experiment (33 dpi).
