# Supplementary material for: The transcriptome of Pinus pinaster under Fusarium circinatum challenge
Source: BMC Genomics. 2020 Jan 8;21:28. doi: 10.1186/s12864-019-6444-0 (PMC6950806; doi:10.1186/s12864-019-6444-0)
Supplement: Supplementary file 2 — Additional file 2. Statistics for each TransABySS and Trinity assembly. N seq: number of transcripts; N bases: number of bases; Mean length: mean length of the transcripts; N50: N50 value; Ns: number of unknown bases; % GC: guanine and cytosine content; trinity-N: in silico normalized trinity assembly; trinity-nN: non-normalized trinity assemblies. * Best quality preliminary assemblies selected to generate the final assembly. [file 12864_2019_6444_MOESM2_ESM.pdf]

Additional file 2: Statistics for each TransABySS and Trinity assembly. N seq: number of transcripts; N bases: number of bases; Mean length: mean length of the transcripts; N50: N50 value; Ns: number of unknown bases; % GC: guanine and cytosine content; trinity-N: *in silico* normalized trinity assembly; trinity-nN: non-normalized trinity assemblies. * Best quality preliminary assemblies selected to generate the final assembly.

Sofware	kmer	N seq	N bases	Mean length	N50	Ns	% GC
**transABySS**	21	155,002	130,062,963	839.11	987	0	42
**transABySS**	25	177,632	156,537,679	881.25	1073	0	42
**transABySS**	29	194,496	171,614,178	882.35	1077	0	42
**transABySS ***	37	216,505	191,809,279	885.93	1082	0	42
**transABySS ***	45	175,922	157,983,806	898.03	1149	0	42
**transABySS ***	53	227,127	200,483,638	882.69	1067	0	42
**transABySS ***	61	216,262	192,122,235	888.38	1065	0	42
**transABySS ***	69	185,869	168,685,723	907.55	1094	0	43
**transABySS ***	77	123,114	116,609,159	947.16	1164	0	43
**trinity-N ***	19	220,217	218,692,491	993.08	1248	0	42
**trinity-N ***	21	221,484	306,982,861	1386.03	2013	0	42
**trinity-N ***	23	222,996	324,494,700	1455.16	2117	0	42
**trinity-N ***	25	254,226	338,343,159	1330.88	1939	0	42
**trinity-N ***	27	251,125	339,266,456	1350.99	1964	0	42
**trinity-N ***	29	245,476	338,191,077	1377.7	1997	0	42
**trinity-N ***	31	240,841	337,312,739	1400.56	2030	0	42
**trinity-nN ***	19	192,124	187,315,834	974.97	1246	0	42
**trinity-nN ***	21	193,012	227,145,680	1176.85	1681	0	42
**trinity-nN ***	23	194,285	234,918,497	1209.14	1754	0	42
**trinity-nN ***	25	214,734	247,127,783	1150.86	1647	0	42
**trinity-nN ***	27	213,152	249,048,800	1168.41	1686	0	42
**Merged assembly**	**19-77**	**49,624**	**68,941,872**	**1389.28**	**1861**	**0**	**45**
