# Supplementary material for: The transcriptome of Pinus pinaster under Fusarium circinatum challenge
Source: BMC Genomics. 2020 Jan 8;21:28. doi: 10.1186/s12864-019-6444-0 (PMC6950806; doi:10.1186/s12864-019-6444-0)
Supplement: Supplementary file 3 — Additional file 3. Comparative statistics between normalized (Norm) and non-normalized (N-norm) Trinity preliminary assemblies. Kmer value; % of mapped fragments; % of good mapping; AS: assembly score; OP: optimal score; OC: optimal cutoff; Number of good contigs; % good contigs. [file 12864_2019_6444_MOESM3_ESM.pdf]

Additional file 3: Comparative statistics between normalized (Norm) and non-normalized (N-norm) Trinity preliminary assemblies. Kmer value; % of mapped fragments; % of good mapping; AS: assembly score; OP: optimal score; OC: optimal cutoff; Number of good contigs; % good contigs.

	kmer	% mapped fragm	% good map	AS	OP	OC	N. good contigs	% good contigs
**Norm**	19	89	71	0.204	0.276	0.105	183,664	83
**N-norm**	19	93	76	0.252	0.334	0.15	161,595	84
**Norm**	21	92	76	0.211	0.330	0.124	179,704	81
**N-norm**	21	93	77	0.257	0.360	0.185	158,473	81
**Norm**	23	92	78	0.219	0.345	0.127	181,600	81
**N-norm**	23	94	77	0.263	0.366	0.191	159,712	82
**Norm**	25	91	75	0.204	0.326	0.133	202,702	80
**N-norm**	25	93	76	0.24	0.365	0.239	164,395	77
**Norm**	27	91	76	0.20	0.334	0.159	196,564	78
**N-norm**	27	93	77	0.244	0.371	0.222	166,792	78
