# Supplementary material for: The transcriptome of Pinus pinaster under Fusarium circinatum challenge
Source: BMC Genomics. 2020 Jan 8;21:28. doi: 10.1186/s12864-019-6444-0 (PMC6950806; doi:10.1186/s12864-019-6444-0)

Additional file 4: BUSCO analysis against the embryophyta lineage database comparing the last *Pinus de novo* transcriptomes published. *P. patula* v1.0 (Visser et al. 2015); *P. patula* v2.0 and *P. tecunumanii* (Visser et al. 2018).

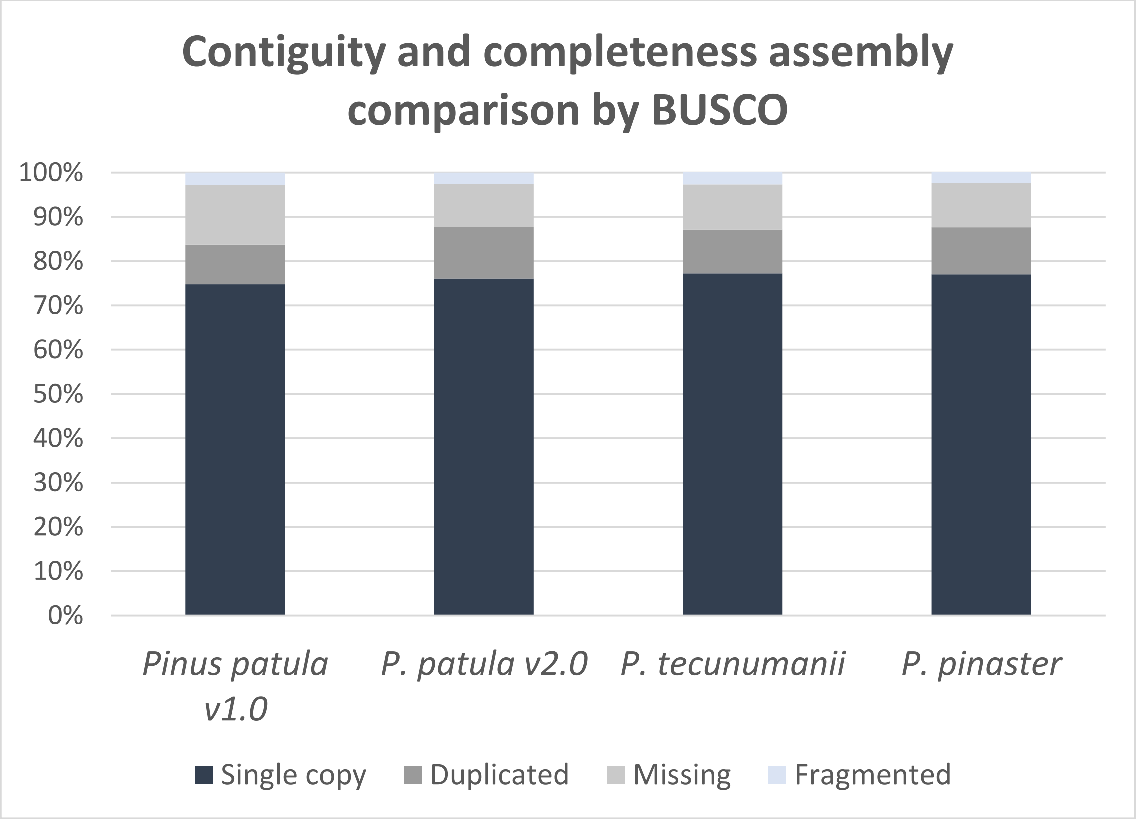

Supplement: Supplementary file 4 — Additional file 4. BUSCO analysis against the embryophyta lineage database comparing the last Pinus de novo transcriptomes published. P. patula v1.0 [110]; P. patula v2.0 and P. tecunumanii [108]. [file 12864_2019_6444_MOESM4_ESM.pdf]
