# Supplementary material for: The transcriptome of Pinus pinaster under Fusarium circinatum challenge
Source: BMC Genomics. 2020 Jan 8;21:28. doi: 10.1186/s12864-019-6444-0 (PMC6950806; doi:10.1186/s12864-019-6444-0)

Additional file 7: Principal component analyses (PCA) for *Pinus pinaster* (above) and *Fusarium circinatum* (below) rlog data of the differential expression gene analysis (DESeq2). In red: mock-inoculated samples; in blue: inoculated samples at 3 dpi; in green: inoculated samples at 5 dpi; in yellow: inoculated samples at 10 dpi.

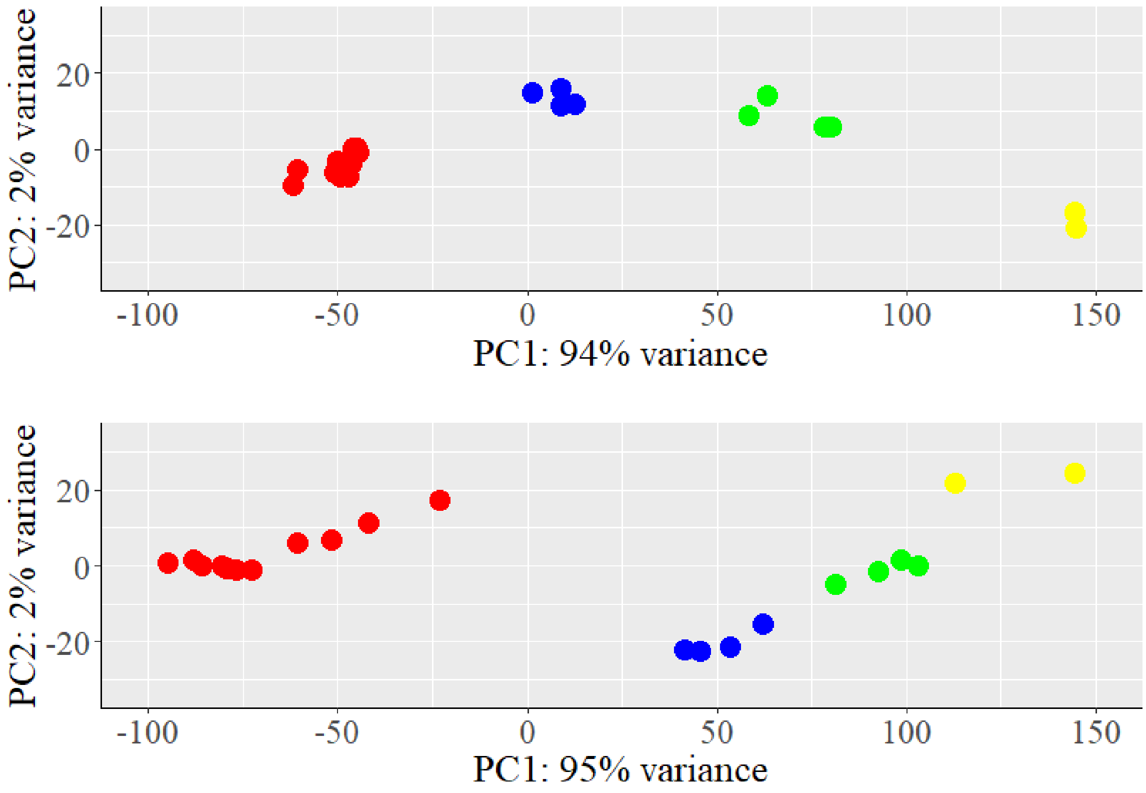

Supplement: Supplementary file 8 — Additional file 8. Principal component analyses (PCA) for Pinus pinaster (above) and Fusarium circinatum (below) rlog data of the differential expression gene analysis (DESeq2). In red: mock-inoculated samples; in blue: inoculated samples at 3 dpi; in green: inoculated samples at 5 dpi; in yellow: inoculated samples at 10 dpi. [file 12864_2019_6444_MOESM8_ESM.pdf]
