# Supplementary material for: The transcriptome of Pinus pinaster under Fusarium circinatum challenge
Source: BMC Genomics. 2020 Jan 8;21:28. doi: 10.1186/s12864-019-6444-0 (PMC6950806; doi:10.1186/s12864-019-6444-0)
Supplement: Supplementary file 10 — Additional file 10. Significantly enriched GO terms identified from Pinus pinaster genes in each cluster. [file 12864_2019_6444_MOESM10_ESM.pdf]

Additional file 10: Significantly enriched GO terms identified from *Pinus pinaster* genes in each cluster.

category	numDEInCat	numInCat	term	ontology	FDR overrepresented p-value
CLUSTER 1					
GO:1901700	134	1295	response to oxygen-containing compound	BP	1,85E-12
GO:0010033	132	1366	response to organic substance	BP	4,55E-10
GO:0010200	24	79	response to chitin	BP	5,49E-10
GO:0042221	174	2025	response to chemical	BP	5,49E-10
GO:0050896	255	3469	response to stimulus	BP	1,12E-08
GO:0009719	100	1030	response to endogenous stimulus	BP	2,16E-07
GO:0010243	28	144	response to organonitrogen compound	BP	2,26E-07
GO:0009751	35	211	response to salicylic acid	BP	5,92E-07
GO:0016298	22	111	lipase activity	MF	5,08E-05
GO:0009753	31	203	response to jasmonic acid	BP	5,08E-05
GO:0016740	91	983	transferase activity	MF	7,66E-05
GO:0010618	15	56	aerenchyma formation	BP	7,66E-05
GO:008016	24	144	response to karrikin	BP	8,17E-05

7					
GO:0003824	208	2861	catalytic activity	MF	8,47E-05
GO:0009725	84	937	response to hormone	BP	0,00010917
GO:0071944	181	2502	cell periphery	CC	0,000122859
GO:0005516	16	66	calmodulin binding	MF	0,000125776
GO:1901698	30	212	response to nitrogen compound	BP	0,000128386
GO:0010310	15	61	regulation of hydrogen peroxide metabolic process	BP	0,0001685
GO:0006950	150	2044	response to stress	BP	0,000265499
GO:0002213	16	76	defense response to insect	BP	0,000265499
GO:0071446	18	90	cellular response to salicylic acid stimulus	BP	0,000265499
GO:2000377	17	82	regulation of reactive oxygen species metabolic process	BP	0,000338558
GO:0010942	16	76	positive regulation of cell death	BP	0,000406023
GO:0009863	17	85	salicylic acid mediated signaling pathway	BP	0,000513296
GO:0009611	31	236	response to wounding	BP	0,000528085
GO:0014070	41	366	response to organic cyclic compound	BP	0,000658126
GO:003134	16	80	negative regulation of defense response	BP	0,000693624

8					
GO:0009873	11	39	ethylene-activated signaling pathway	BP	0,000693624
GO:0009862	15	70	systemic acquired resistance, salicylic acid mediated signaling pathway	BP	0,000693624
GO:0009723	22	139	response to ethylene	BP	0,000693624
GO:0080134	28	208	regulation of response to stress	BP	0,000724346
GO:0071369	12	47	cellular response to ethylene stimulus	BP	0,00077844
GO:0009816	17	89	defense response to bacterium, incompatible interaction	BP	0,000859615
GO:0010150	17	90	leaf senescence	BP	0,00086184
GO:0031347	24	167	regulation of defense response	BP	0,001119854
GO:0010260	17	92	animal organ senescence	BP	0,001119854
GO:0009625	16	88	response to insect	BP	0,00134899
GO:0016829	21	135	lyase activity	MF	0,00223238
GO:0009627	17	101	systemic acquired resistance	BP	0,002233913
GO:0005886	145	1989	plasma membrane	CC	0,002241834
GO:0003674	317	5050	molecular_function	MF	0,00277698
GO:000016	11	47	phosphorelay signal transduction system	BP	0,003135811

0					
GO:0044711	47	463	NA	NA	0,003640324
GO:0001666	16	95	response to hypoxia	BP	0,00403548
GO:0016757	26	202	transferase activity, transferring glycosyl groups	MF	0,005217769
GO:0043436	54	565	oxoacid metabolic process	BP	0,005556955
GO:0006082	54	566	organic acid metabolic process	BP	0,005628029
GO:0036293	16	99	response to decreased oxygen levels	BP	0,00586263
GO:0070482	16	99	response to oxygen levels	BP	0,00586263
GO:0019752	53	552	carboxylic acid metabolic process	BP	0,005869834
GO:0016846	7	18	carbon-sulfur lyase activity	MF	0,00646179
GO:0010193	10	42	response to ozone	BP	0,009681103
CLUSTER 2					
GO:0005829	495	2023	cytosol	CC	6,99E-43
GO:0044238	595	2915	primary metabolic process	BP	8,02E-36
GO:0032991	339	1299	protein-containing complex	CC	1,00E-32
GO:0071704	637	3250	organic substance metabolic process	BP	1,41E-31

GO:0043234	245	878	NA	NA	3,37E-30
GO:0044237	639	3343	cellular metabolic process	BP	1,44E-28
GO:0008152	752	4137	metabolic process	BP	5,56E-26
GO:0009987	856	4934	cellular process	BP	7,30E-26
GO:0051641	147	445	cellular localization	BP	9,28E-26
GO:0009056	218	816	catabolic process	BP	4,51E-25
GO:0051649	131	385	establishment of localization in cell	BP	1,17E-23
GO:0046907	125	355	intracellular transport	BP	1,41E-23
GO:0043581	70	128	NA	NA	3,79E-23
GO:1901564	205	756	organonitrogen compound metabolic process	BP	3,79E-23
GO:1901575	197	744	organic substance catabolic process	BP	1,04E-22
GO:0044248	189	697	cellular catabolic process	BP	1,27E-22
GO:0044085	162	524	cellular component biogenesis	BP	1,27E-22
GO:0006807	327	1517	nitrogen compound metabolic process	BP	1,33E-22
GO:0003674	869	5050	molecular_function	MF	2,78E-22

GO:0010467	175	610	gene expression	BP	4,36E-22
GO:0005488	333	1499	binding	MF	6,08E-22
GO:0022613	90	218	ribonucleoprotein complex biogenesis	BP	1,12E-20
GO:0008150	1174	7614	biological_process	BP	1,19E-19
GO:0016482	96	266	cytosolic transport	BP	1,19E-19
GO:0044260	366	1837	cellular macromolecule metabolic process	BP	1,19E-19
GO:0044281	253	1074	small molecule metabolic process	BP	4,53E-19
GO:0034641	280	1319	cellular nitrogen compound metabolic process	BP	4,90E-19
GO:0016192	69	160	vesicle-mediated transport	BP	8,57E-19
GO:0043170	380	1940	macromolecule metabolic process	BP	9,52E-19
GO:0048193	39	60	Golgi vesicle transport	BP	3,17E-18
GO:0019538	268	1189	protein metabolic process	BP	1,36E-17
GO:0044267	258	1139	cellular protein metabolic process	BP	2,74E-17
GO:0006139	237	1123	nucleobase-containing compound metabolic process	BP	4,72E-17
GO:0002181	72	161	cytoplasmic translation	BP	1,22E-16

GO:0071840	291	1402	cellular component organization or biogenesis	BP	3,29E-16
GO:0055086	110	389	nucleobase-containing small molecule metabolic process	BP	8,10E-16
GO:0044710	341	1649	NA	NA	1,14E-15
GO:0043933	97	296	protein-containing complex subunit organization	BP	2,11E-15
GO:0003824	516	2861	catalytic activity	MF	2,12E-15
GO:0046483	262	1288	heterocycle metabolic process	BP	2,55E-15
GO:0008104	89	270	protein localization	BP	4,46E-15
GO:0033036	109	373	macromolecule localization	BP	9,24E-15
GO:0006725	272	1357	cellular aromatic compound metabolic process	BP	1,07E-14
GO:0007346	42	76	regulation of mitotic cell cycle	BP	1,07E-14
GO:0022607	94	290	cellular component assembly	BP	1,07E-14
GO:0034645	138	511	cellular macromolecule biosynthetic process	BP	2,06E-14
GO:0042254	72	188	ribosome biogenesis	BP	4,46E-14
GO:0070727	87	274	cellular macromolecule localization	BP	5,38E-14
GO:0034613	82	249	cellular protein localization	BP	5,40E-14

GO:0034622	63	160	cellular protein-containing complex assembly	BP	9,14E-14
GO:0000324	23	30	fungal-type vacuole	CC	1,42E-13
GO:0043254	24	33	regulation of protein complex assembly	BP	1,78E-13
GO:0006508	59	151	proteolysis	BP	1,87E-13
GO:0065003	71	196	protein-containing complex assembly	BP	2,06E-13
GO:1901360	282	1443	organic cyclic compound metabolic process	BP	2,18E-13
GO:0006753	98	359	nucleoside phosphate metabolic process	BP	2,91E-13
GO:0043632	52	127	modification-dependent macromolecule catabolic process	BP	7,51E-13
GO:0051603	53	131	proteolysis involved in cellular protein catabolic process	BP	8,25E-13
GO:0032153	25	35	cell division site	CC	8,82E-13
GO:0033043	39	85	regulation of organelle organization	BP	9,50E-13
GO:0009059	139	545	macromolecule biosynthetic process	BP	1,02E-12
GO:0000329	18	21	fungal-type vacuole membrane	CC	1,02E-12
GO:0017076	69	199	purine nucleotide binding	MF	1,02E-12
GO:0043161	37	69	proteasome-mediated ubiquitin-dependent protein catabolic process	BP	1,20E-12

GO:0009117	96	357	nucleotide metabolic process	BP	1,21E-12
GO:0030684	37	67	preribosome	CC	1,21E-12
GO:1901363	180	797	heterocyclic compound binding	MF	1,22E-12
GO:0005515	111	421	protein binding	MF	1,33E-12
GO:0097159	180	800	organic cyclic compound binding	MF	1,49E-12
GO:0001882	68	197	nucleoside binding	MF	1,87E-12
GO:0001883	68	197	purine nucleoside binding	MF	1,87E-12
GO:0032549	68	197	ribonucleoside binding	MF	1,87E-12
GO:0032550	68	197	purine ribonucleoside binding	MF	1,87E-12
GO:0032555	68	197	purine ribonucleotide binding	MF	1,87E-12
GO:0002183	17	18	cytoplasmic translational initiation	BP	1,91E-12
GO:0010498	37	70	proteasomal protein catabolic process	BP	1,92E-12
GO:0051640	31	57	organelle localization	BP	1,92E-12
GO:0044257	53	135	cellular protein catabolic process	BP	2,02E-12
GO:0072521	86	316	purine-containing compound metabolic process	BP	2,58E-12

GO:0006520	82	250	cellular amino acid metabolic process	BP	2,72E-12
GO:0019637	117	470	organophosphate metabolic process	BP	3,21E-12
GO:0032553	68	200	ribonucleotide binding	MF	3,63E-12
GO:0019941	50	124	modification-dependent protein catabolic process	BP	3,75E-12
GO:0051726	63	176	regulation of cell cycle	BP	4,65E-12
GO:0035639	66	191	purine ribonucleoside triphosphate binding	MF	4,94E-12
GO:0044249	254	1189	cellular biosynthetic process	BP	4,94E-12
GO:0006996	157	714	organelle organization	BP	5,06E-12
GO:0034660	69	202	ncRNA metabolic process	BP	5,14E-12
GO:0051128	57	162	regulation of cellular component organization	BP	6,97E-12
GO:0051716	234	1161	cellular response to stimulus	BP	8,36E-12
GO:0043168	87	294	anion binding	MF	9,83E-12
GO:0006511	49	123	ubiquitin-dependent protein catabolic process	BP	9,94E-12
GO:0006888	24	35	ER to Golgi vesicle-mediated transport	BP	1,07E-11
GO:0044265	65	195	cellular macromolecule catabolic process	BP	1,14E-11

GO:0033554	124	503	cellular response to stress	BP	2,41E-11
GO:0000322	24	37	storage vacuole	CC	2,53E-11
GO:0072523	72	264	purine-containing compound catabolic process	BP	3,86E-11
GO:0006913	39	91	nucleocytoplasmic transport	BP	4,61E-11
GO:0051169	39	91	nuclear transport	BP	4,61E-11
GO:0051656	27	50	establishment of organelle localization	BP	6,81E-11
GO:0006152	71	261	purine nucleoside catabolic process	BP	7,20E-11
GO:0051168	32	65	nuclear export	BP	7,90E-11
GO:0034655	85	330	nucleobase-containing compound catabolic process	BP	7,96E-11
GO:0044087	32	63	regulation of cellular component biogenesis	BP	7,96E-11
GO:0030163	56	159	protein catabolic process	BP	8,08E-11
GO:0044763	503	3000	NA	NA	9,77E-11
GO:0000323	29	53	lytic vacuole	CC	9,78E-11
GO:0006886	68	215	intracellular protein transport	BP	1,06E-10
GO:1901576	263	1270	organic substance biosynthetic process	BP	1,09E-10

GO:0006163	78	295	purine nucleotide metabolic process	BP	1,12E-10
GO:0042278	77	290	purine nucleoside metabolic process	BP	1,23E-10
GO:0009141	71	264	nucleoside triphosphate metabolic process	BP	1,28E-10
GO:0009164	71	264	nucleoside catabolic process	BP	1,43E-10
GO:0042454	71	264	ribonucleoside catabolic process	BP	1,43E-10
GO:0046130	70	260	purine ribonucleoside catabolic process	BP	1,49E-10
GO:0015031	70	226	protein transport	BP	1,50E-10
GO:0045184	70	226	establishment of protein localization	BP	1,50E-10
GO:0016043	236	1214	cellular component organization	BP	1,52E-10
GO:1901292	70	259	nucleoside phosphate catabolic process	BP	1,53E-10
GO:0009143	69	255	nucleoside triphosphate catabolic process	BP	1,53E-10
GO:0009146	69	255	purine nucleoside triphosphate catabolic process	BP	1,53E-10
GO:0019693	77	291	ribose phosphate metabolic process	BP	1,63E-10
GO:0000166	72	237	nucleotide binding	MF	1,73E-10
GO:1901265	72	237	nucleoside phosphate binding	MF	1,73E-10

GO:0009119	78	297	ribonucleoside metabolic process	BP	1,76E-10
GO:0009199	70	261	ribonucleoside triphosphate metabolic process	BP	1,98E-10
GO:0009144	70	261	purine nucleoside triphosphate metabolic process	BP	2,00E-10
GO:0009116	78	298	nucleoside metabolic process	BP	2,15E-10
GO:0030117	13	15	membrane coat	CC	2,15E-10
GO:0030120	13	15	vesicle coat	CC	2,15E-10
GO:0048475	13	15	coated membrane	CC	2,15E-10
GO:1901565	95	381	organonitrogen compound catabolic process	BP	2,16E-10
GO:0030427	21	30	site of polarized growth	CC	2,18E-10
GO:0046128	76	289	purine ribonucleoside metabolic process	BP	2,27E-10
GO:0009058	276	1361	biosynthetic process	BP	2,27E-10
GO:0009150	75	285	purine ribonucleotide metabolic process	BP	2,79E-10
GO:0043167	134	546	ion binding	MF	2,83E-10
GO:0046700	91	368	heterocycle catabolic process	BP	2,92E-10
GO:0009166	69	258	nucleotide catabolic process	BP	2,92E-10

GO:0006195	68	254	purine nucleotide catabolic process	BP	2,92E-10
GO:0009154	68	254	purine ribonucleotide catabolic process	BP	2,92E-10
GO:0009203	68	254	ribonucleoside triphosphate catabolic process	BP	2,92E-10
GO:0009207	68	254	purine ribonucleoside triphosphate catabolic process	BP	2,92E-10
GO:0009261	68	254	ribonucleotide catabolic process	BP	2,92E-10
GO:0009259	76	290	ribonucleotide metabolic process	BP	2,97E-10
GO:0010564	41	103	regulation of cell cycle process	BP	3,84E-10
GO:0009205	69	260	purine ribonucleoside triphosphate metabolic process	BP	3,87E-10
GO:1901135	111	473	carbohydrate derivative metabolic process	BP	3,99E-10
GO:0044270	91	370	cellular nitrogen compound catabolic process	BP	4,15E-10
GO:0030529	127	479	NA	NA	4,20E-10
GO:0006412	81	256	translation	BP	6,11E-10
GO:0036094	74	253	small molecule binding	MF	6,37E-10
GO:0045786	20	29	negative regulation of cell cycle	BP	6,47E-10
GO:0006793	190	965	phosphorus metabolic process	BP	8,20E-10

GO:1901361	92	380	organic cyclic compound catabolic process	BP	8,36E-10
GO:0000070	18	26	mitotic sister chromatid segregation	BP	9,14E-10
GO:0051179	216	1096	localization	BP	1,07E-09
GO:0006357	32	67	regulation of transcription by RNA polymerase II	BP	1,07E-09
GO:0003723	76	272	RNA binding	MF	1,10E-09
GO:0032505	22	34	reproduction of a single-celled organism	BP	1,20E-09
GO:0016072	50	134	rRNA metabolic process	BP	1,40E-09
GO:0046434	70	271	organophosphate catabolic process	BP	1,43E-09
GO:0019439	89	369	aromatic compound catabolic process	BP	1,55E-09
GO:0007163	23	38	establishment or maintenance of cell polarity	BP	1,81E-09
GO:0016070	115	522	RNA metabolic process	BP	2,27E-09
GO:0009057	72	257	macromolecule catabolic process	BP	2,43E-09
GO:0006364	49	132	rRNA processing	BP	2,47E-09
GO:0006796	186	954	phosphate-containing compound metabolic process	BP	2,49E-09
GO:0005681	22	36	spliceosomal complex	CC	3,05E-09

GO:0030686	27	50	90S preribosome	CC	3,05E-09
GO:0017111	68	270	nucleoside-triphosphatase activity	MF	3,23E-09
GO:0005933	13	14	cellular bud	CC	3,89E-09
GO:0019752	132	552	carboxylic acid metabolic process	BP	3,95E-09
GO:0045292	20	31	mRNA cis splicing, via spliceosome	BP	4,14E-09
GO:0071585	15	18	detoxification of cadmium ion	BP	4,34E-09
GO:1990170	15	18	stress response to cadmium ion	BP	4,34E-09
GO:0007049	83	329	cell cycle	BP	4,34E-09
GO:0022402	78	300	cell cycle process	BP	5,12E-09
GO:1901136	74	296	carbohydrate derivative catabolic process	BP	5,16E-09
GO:0006366	22	40	transcription by RNA polymerase II	BP	5,48E-09
GO:0043934	22	35	sporulation	BP	5,83E-09
GO:0043436	133	565	oxoacid metabolic process	BP	6,89E-09
GO:0006082	133	566	organic acid metabolic process	BP	7,88E-09
GO:0016462	72	294	pyrophosphatase activity	MF	8,01E-09

GO:0016818	72	294	hydrolase activity, acting on acid anhydrides, in phosphorus-containing anhydrides	MF	8,01E-09
GO:0005634	550	3326	nucleus	CC	8,37E-09
GO:0022413	20	31	reproductive process in single-celled organism	BP	9,43E-09
GO:0016817	73	300	hydrolase activity, acting on acid anhydrides	MF	9,80E-09
GO:0097501	15	19	stress response to metal ion	BP	1,02E-08
GO:0071276	14	17	cellular response to cadmium ion	BP	1,16E-08
GO:0005935	11	11	cellular bud neck	CC	1,22E-08
GO:0051234	201	1031	establishment of localization	BP	1,32E-08
GO:0071826	31	67	ribonucleoprotein complex subunit organization	BP	1,36E-08
GO:1901658	72	292	glycosyl compound catabolic process	BP	1,44E-08
GO:1901657	82	341	glycosyl compound metabolic process	BP	1,52E-08
GO:0005934	11	11	cellular bud tip	CC	1,53E-08
GO:0000280	24	52	nuclear division	BP	1,73E-08
GO:0000819	18	30	sister chromatid segregation	BP	1,78E-08
GO:0006810	199	1019	transport	BP	2,08E-08

GO:0044699	630	4159	NA	NA	2,11E-08
GO:0000460	19	30	maturation of 5.8S rRNA	BP	2,15E-08
GO:0000466	19	30	maturation of 5.8S rRNA from tricistronic rRNA transcript (SSU-rRNA, 5.8S rRNA, LSU-rRNA)	BP	2,15E-08
GO:0010948	30	67	negative regulation of cell cycle process	BP	2,58E-08
GO:0006406	18	30	mRNA export from nucleus	BP	3,01E-08
GO:0051028	18	30	mRNA transport	BP	3,01E-08
GO:0007067	23	49	NA	NA	3,54E-08
GO:0006405	25	52	RNA export from nucleus	BP	3,76E-08
GO:0042277	16	24	peptide binding	MF	4,74E-08
GO:0090304	153	805	nucleic acid metabolic process	BP	5,94E-08
GO:0006396	82	336	RNA processing	BP	6,17E-08
GO:0050657	25	53	nucleic acid transport	BP	6,65E-08
GO:0050658	25	53	RNA transport	BP	6,65E-08
GO:0051236	25	53	establishment of RNA localization	BP	6,65E-08
GO:0012505	116	524	endomembrane system	CC	6,72E-08

GO:0022618	29	64	ribonucleoprotein complex assembly	BP	8,75E-08
GO:0033218	16	25	amide binding	MF	8,84E-08
GO:0005525	28	64	GTP binding	MF	9,05E-08
GO:0019001	28	64	guanyl nucleotide binding	MF	9,05E-08
GO:0032561	28	64	guanyl ribonucleotide binding	MF	9,05E-08
GO:0070316	10	10	regulation of G0 to G1 transition	BP	9,20E-08
GO:0070317	10	10	negative regulation of G0 to G1 transition	BP	9,20E-08
GO:0009126	54	216	purine nucleoside monophosphate metabolic process	BP	9,88E-08
GO:0030134	12	14	COPII-coated ER to Golgi transport vesicle	CC	1,02E-07
GO:0043562	18	29	cellular response to nitrogen levels	BP	1,04E-07
GO:0006403	25	54	RNA localization	BP	1,10E-07
GO:0003676	117	549	nucleic acid binding	MF	1,11E-07
GO:0006995	16	24	cellular response to nitrogen starvation	BP	1,29E-07
GO:0030435	19	31	sporulation resulting in formation of a cellular spore	BP	1,55E-07
GO:0048523	94	412	negative regulation of cellular process	BP	1,63E-07

GO:0030532	17	27	small nuclear ribonucleoprotein complex	CC	1,98E-07
GO:0019318	27	57	hexose metabolic process	BP	1,98E-07
GO:0006950	360	2044	response to stress	BP	2,05E-07
GO:0009167	53	215	purine ribonucleoside monophosphate metabolic process	BP	2,05E-07
GO:0006351	26	66	transcription, DNA-templated	BP	2,05E-07
GO:0044429	79	287	mitochondrial part	CC	2,10E-07
GO:0050794	351	2057	regulation of cellular process	BP	2,26E-07
GO:0034470	53	170	ncRNA processing	BP	2,45E-07
GO:0009894	27	61	regulation of catabolic process	BP	2,83E-07
GO:0009123	54	221	nucleoside monophosphate metabolic process	BP	2,84E-07
GO:0009161	53	217	ribonucleoside monophosphate metabolic process	BP	3,14E-07
GO:0044425	163	794	membrane part	CC	3,60E-07
GO:0008652	44	127	cellular amino acid biosynthetic process	BP	3,76E-07
GO:0030864	11	12	cortical actin cytoskeleton	CC	3,76E-07
GO:0005048	9	10	signal sequence binding	MF	3,78E-07

GO:0016787	179	962	hydrolase activity	MF	4,06E-07
GO:0051336	26	56	regulation of hydrolase activity	BP	4,13E-07
GO:0061025	17	28	membrane fusion	BP	4,19E-07
GO:0009636	22	46	response to toxic substance	BP	6,14E-07
GO:0051130	24	56	positive regulation of cellular component organization	BP	6,24E-07
GO:0071852	14	20	fungal-type cell wall organization or biogenesis	BP	6,47E-07
GO:0042274	32	78	ribosomal small subunit biogenesis	BP	6,62E-07
GO:0044452	15	23	nucleolar part	CC	6,79E-07
GO:0030490	29	67	maturation of SSU-rRNA	BP	7,33E-07
GO:0005816	17	30	spindle pole body	CC	7,57E-07
GO:0006200	48	199	NA	NA	7,67E-07
GO:0009125	48	199	nucleoside monophosphate catabolic process	BP	7,67E-07
GO:0009128	48	199	purine nucleoside monophosphate catabolic process	BP	7,67E-07
GO:0009158	48	199	ribonucleoside monophosphate catabolic process	BP	7,67E-07
GO:0009169	48	199	purine ribonucleoside monophosphate catabolic process	BP	7,67E-07

GO:0016887	48	199	ATPase activity	MF	7,67E-07
GO:0046034	49	204	ATP metabolic process	BP	7,75E-07
GO:0031597	10	11	cytosolic proteasome complex	CC	8,19E-07
GO:0034515	10	11	proteasome storage granule	CC	8,19E-07
GO:0005937	10	11	mating projection	CC	8,75E-07
GO:1901566	73	270	organonitrogen compound biosynthetic process	BP	9,09E-07
GO:0031974	181	917	membrane-enclosed lumen	CC	9,88E-07
GO:0034293	17	28	sexual sporulation	BP	9,95E-07
GO:0043935	17	28	sexual sporulation resulting in formation of a cellular spore	BP	9,95E-07
GO:0042144	8	8	vacuole fusion, non-autophagic	BP	1,35E-06
GO:0000462	27	62	maturation of SSU-rRNA from tricistronic rRNA transcript (SSU-rRNA, 5.8S rRNA, LSU-rRNA)	BP	1,62E-06
GO:0016050	16	28	vesicle organization	BP	1,72E-06
GO:0000398	27	66	mRNA splicing, via spliceosome	BP	1,82E-06
GO:0006360	7	7	transcription by RNA polymerase I	BP	1,91E-06
GO:0030479	10	11	actin cortical patch	CC	1,93E-06

GO:0044428	174	900	nuclear part	CC	2,09E-06
GO:0065007	423	2605	biological regulation	BP	2,27E-06
GO:0070013	176	900	intracellular organelle lumen	CC	2,29E-06
GO:0071702	116	532	organic substance transport	BP	2,30E-06
GO:0019320	17	30	hexose catabolic process	BP	2,65E-06
GO:0046365	17	30	monosaccharide catabolic process	BP	2,65E-06
GO:0043233	176	902	organelle lumen	CC	2,69E-06
GO:0007034	21	49	vacuolar transport	BP	2,86E-06
GO:0030447	11	14	filamentous growth	BP	3,09E-06
GO:0007010	46	157	cytoskeleton organization	BP	3,10E-06
GO:0032889	8	8	regulation of vacuole fusion, non-autophagic	BP	3,12E-06
GO:0030662	16	37	coated vesicle membrane	CC	3,32E-06
GO:0006418	13	21	tRNA aminoacylation for protein translation	BP	3,32E-06
GO:0043038	13	21	amino acid activation	BP	3,32E-06
GO:0043039	13	21	tRNA aminoacylation	BP	3,32E-06

GO:0000967	9	11	rRNA 5'-end processing	BP	3,62E-06
GO:0034471	9	11	ncRNA 5'-end processing	BP	3,62E-06
GO:0030437	16	27	ascospore formation	BP	3,64E-06
GO:0050896	559	3469	response to stimulus	BP	4,03E-06
GO:0060260	8	9	regulation of transcription initiation from RNA polymerase II promoter	BP	4,19E-06
GO:2000142	8	9	regulation of DNA-templated transcription, initiation	BP	4,19E-06
GO:0071822	59	222	NA	NA	4,24E-06
GO:0071004	10	12	U2-type prespliceosome	CC	4,33E-06
GO:0071010	10	12	prespliceosome	CC	4,33E-06
GO:0000375	27	70	RNA splicing, via transesterification reactions	BP	4,35E-06
GO:0000377	27	70	RNA splicing, via transesterification reactions with bulged adenosine as nucleophile	BP	4,35E-06
GO:0046540	9	10	U4/U6 x U5 tri-snRNP complex	CC	4,55E-06
GO:0000245	11	15	spliceosomal complex assembly	BP	6,30E-06
GO:0061024	35	107	membrane organization	BP	6,86E-06
GO:0030554	41	136	adenyl nucleotide binding	MF	7,19E-06

GO:0030135	22	62	coated vesicle	CC	7,23E-06
GO:0005684	13	20	U2-type spliceosomal complex	CC	7,35E-06
GO:0005815	18	37	microtubule organizing center	CC	7,66E-06
GO:0050789	395	2437	regulation of biological process	BP	7,91E-06
GO:0071540	7	7	eukaryotic translation initiation factor 3 complex, eIF3e	CC	7,93E-06
GO:0032040	14	23	small-subunit processome	CC	7,93E-06
GO:0006006	15	25	glucose metabolic process	BP	8,06E-06
GO:0044271	68	279	cellular nitrogen compound biosynthetic process	BP	8,08E-06
GO:0031329	23	54	regulation of cellular catabolic process	BP	8,08E-06
GO:0031669	39	131	cellular response to nutrient levels	BP	8,87E-06
GO:0051082	15	25	unfolded protein binding	MF	8,90E-06
GO:0010243	44	144	response to organonitrogen compound	BP	9,50E-06
GO:0030433	11	16	ubiquitin-dependent ERAD pathway	BP	9,73E-06
GO:0005938	19	45	cell cortex	CC	1,02E-05
GO:0048311	8	8	mitochondrion distribution	BP	1,02E-05

GO:0051646	8	8	mitochondrion localization	BP	1,02E-05
GO:0042273	20	42	ribosomal large subunit biogenesis	BP	1,13E-05
GO:0006007	13	20	glucose catabolic process	BP	1,13E-05
GO:0004568	8	8	chitinase activity	MF	1,15E-05
GO:0006892	13	21	post-Golgi vesicle-mediated transport	BP	1,27E-05
GO:0006457	26	65	protein folding	BP	1,27E-05
GO:0032559	40	134	adenyl ribonucleotide binding	MF	1,32E-05
GO:0051286	13	20	cell tip	CC	1,34E-05
GO:0060187	13	20	cell pole	CC	1,34E-05
GO:1901607	35	103	alpha-amino acid biosynthetic process	BP	1,43E-05
GO:0030133	16	33	transport vesicle	CC	1,47E-05
GO:0044448	16	35	cell cortex part	CC	1,59E-05
GO:0031503	8	9	protein-containing complex localization	BP	1,82E-05
GO:0000469	14	25	cleavage involved in rRNA processing	BP	1,85E-05
GO:0032182	11	18	ubiquitin-like protein binding	MF	1,92E-05

GO:0043130	11	18	ubiquitin binding	MF	1,92E-05
GO:0034629	7	7	cellular protein-containing complex localization	BP	1,95E-05
GO:0004812	13	25	aminoacyl-tRNA ligase activity	MF	2,26E-05
GO:0016875	13	25	ligase activity, forming carbon-oxygen bonds	MF	2,26E-05
GO:0016876	13	25	NA	NA	2,26E-05
GO:0044088	8	9	regulation of vacuole organization	BP	2,30E-05
GO:0043332	8	9	mating projection tip	CC	2,35E-05
GO:0030515	9	12	snoRNA binding	MF	2,38E-05
GO:0007264	12	18	small GTPase mediated signal transduction	BP	2,51E-05
GO:0031667	45	168	response to nutrient levels	BP	3,08E-05
GO:0006461	37	120	NA	NA	3,12E-05
GO:0030466	8	10	chromatin silencing at silent mating-type cassette	BP	3,17E-05
GO:0030687	11	16	preribosome, large subunit precursor	CC	3,24E-05
GO:0000472	8	10	endonucleolytic cleavage to generate mature 5'-end of SSU-rRNA from (SSU-rRNA, 5.8S rRNA, LSU-rRNA)	BP	3,31E-05
GO:1901605	49	172	alpha-amino acid metabolic process	BP	3,31E-05

GO:0005524	38	128	ATP binding	MF	3,45E-05
GO:0043623	29	84	NA	NA	3,47E-05
GO:0071963	8	9	establishment or maintenance of cell polarity regulating cell shape	BP	3,56E-05
GO:0090501	14	26	RNA phosphodiester bond hydrolysis	BP	3,60E-05
GO:0032956	11	18	regulation of actin cytoskeleton organization	BP	3,74E-05
GO:0015931	34	106	nucleobase-containing compound transport	BP	3,78E-05
GO:0034654	45	170	nucleobase-containing compound biosynthetic process	BP	3,85E-05
GO:0008061	9	11	chitin binding	MF	4,05E-05
GO:0019953	35	109	sexual reproduction	BP	4,17E-05
GO:0007005	21	52	mitochondrion organization	BP	4,24E-05
GO:0043603	23	57	cellular amide metabolic process	BP	4,89E-05
GO:0006890	7	8	retrograde vesicle-mediated transport, Golgi to ER	BP	5,51E-05
GO:0060627	12	21	regulation of vesicle-mediated transport	BP	5,52E-05
GO:0030036	20	51	actin cytoskeleton organization	BP	5,63E-05
GO:0045333	15	32	cellular respiration	BP	5,86E-05

GO:0007088	11	18	regulation of mitotic nuclear division	BP	5,86E-05
GO:0016021	56	232	integral component of membrane	CC	6,45E-05
GO:0071541	6	6	eukaryotic translation initiation factor 3 complex, eIF3m	CC	6,53E-05
GO:0048308	8	9	organelle inheritance	BP	6,89E-05
GO:0006397	32	104	mRNA processing	BP	7,15E-05
GO:0015629	15	28	actin cytoskeleton	CC	7,41E-05
GO:0097367	10	14	carbohydrate derivative binding	MF	8,56E-05
GO:0030811	17	37	regulation of nucleotide catabolic process	BP	8,81E-05
GO:0033121	17	37	regulation of purine nucleotide catabolic process	BP	8,81E-05
GO:0006096	10	14	glycolytic process	BP	8,81E-05
GO:0070271	37	125	NA	NA	9,20E-05
GO:0042175	61	249	nuclear outer membrane-endoplasmic reticulum membrane network	CC	9,20E-05
GO:0005996	30	87	monosaccharide metabolic process	BP	9,20E-05
GO:0007015	14	30	actin filament organization	BP	9,58E-05
GO:0033365	42	163	protein localization to organelle	BP	9,76E-05

GO:0008360	12	20	regulation of cell shape	BP	0,000102839
GO:0007097	7	8	nuclear migration	BP	0,000106123
GO:0040023	7	8	NA	NA	0,000106123
GO:0051647	7	8	nucleus localization	BP	0,000106123
GO:0048285	24	78	organelle fission	BP	0,000107934
GO:0051493	13	26	regulation of cytoskeleton organization	BP	0,000107934
GO:0031505	9	12	fungal-type cell wall organization	BP	0,000112138
GO:0051783	11	20	regulation of nuclear division	BP	0,000112938
GO:0060341	13	24	regulation of cellular localization	BP	0,00011296
GO:0005838	7	8	proteasome regulatory particle	CC	0,000115715
GO:0022624	7	8	proteasome accessory complex	CC	0,000115715
GO:0071496	43	165	cellular response to external stimulus	BP	0,000115715
GO:0031668	42	158	cellular response to extracellular stimulus	BP	0,000116006
GO:0030029	20	53	actin filament-based process	BP	0,000118986
GO:0034401	5	5	chromatin organization involved in regulation of transcription	BP	0,000119892

GO:0000922	17	40	spindle pole	CC	0,000120161
GO:0007018	7	8	microtubule-based movement	BP	0,000122438
GO:0005740	56	212	mitochondrial envelope	CC	0,000125555
GO:0000502	22	55	proteasome complex	CC	0,000125555
GO:0006140	17	38	regulation of nucleotide metabolic process	BP	0,000134483
GO:0009118	17	38	regulation of nucleoside metabolic process	BP	0,000134483
GO:1900542	17	38	regulation of purine nucleotide metabolic process	BP	0,000134483
GO:0051246	45	171	regulation of protein metabolic process	BP	0,000141737
GO:0006906	11	18	vesicle fusion	BP	0,000144728
GO:0000480	7	9	endonucleolytic cleavage in 5'-ETS of tricistronic rRNA transcript (SSU-rRNA, 5.8S rRNA, LSU-rRNA)	BP	0,00014692
GO:0005737	1032	7138	cytoplasm	CC	0,000153312
GO:0016874	53	228	ligase activity	MF	0,000155163
GO:0032774	26	88	RNA biosynthetic process	BP	0,000166765
GO:0044724	23	65	NA	NA	0,000167931
GO:0000278	35	123	mitotic cell cycle	BP	0,000169194

GO:0072384	6	6	organelle transport along microtubule	BP	0,000174785
GO:0032970	11	20	regulation of actin filament-based process	BP	0,000176393
GO:0000054	11	19	ribosomal subunit export from nucleus	BP	0,000176393
GO:0033750	11	19	ribosome localization	BP	0,000176393
GO:0033753	11	19	NA	NA	0,000176393
GO:0071166	11	19	ribonucleoprotein complex localization	BP	0,000176393
GO:0071426	11	19	ribonucleoprotein complex export from nucleus	BP	0,000176393
GO:0071428	11	19	rRNA-containing ribonucleoprotein complex export from nucleus	BP	0,000176393
GO:0016052	25	77	carbohydrate catabolic process	BP	0,000182254
GO:0032535	16	38	regulation of cellular component size	BP	0,000184218
GO:0008380	28	94	RNA splicing	BP	0,000185182
GO:0006518	14	28	peptide metabolic process	BP	0,000198112
GO:0048519	119	641	negative regulation of biological process	BP	0,000198583
GO:0044432	62	263	endoplasmic reticulum part	CC	0,000198583
GO:0071241	20	52	cellular response to inorganic substance	BP	0,000198613

GO:0007059	21	69	chromosome segregation	BP	0,00020748
GO:0071824	11	21	protein-DNA complex subunit organization	BP	0,000210961
GO:0005777	55	218	peroxisome	CC	0,000210961
GO:0042579	55	218	microbody	CC	0,000210961
GO:2001020	8	12	regulation of response to DNA damage stimulus	BP	0,0002205
GO:0044765	162	901	NA	NA	0,000233098
GO:0045944	16	35	positive regulation of transcription by RNA polymerase II	BP	0,000235742
GO:0050790	50	196	regulation of catalytic activity	BP	0,000238482
GO:0030126	4	4	COPI vesicle coat	CC	0,000239294
GO:0043486	6	7	histone exchange	BP	0,000243717
GO:0008026	11	21	ATP-dependent helicase activity	MF	0,000244517
GO:0070035	11	21	purine NTP-dependent helicase activity	MF	0,000244517
GO:0005832	6	6	chaperonin-containing T-complex	CC	0,000250399
GO:0031072	8	11	heat shock protein binding	MF	0,000267229
GO:0032543	8	11	mitochondrial translation	BP	0,000267697

GO:0043604	13	25	amide biosynthetic process	BP	0,000267868
GO:0005635	25	85	nuclear envelope	CC	0,000279997
GO:0000243	6	6	commitment complex	CC	0,000284995
GO:0030436	7	8	asexual sporulation	BP	0,000289565
GO:0000001	6	6	mitochondrion inheritance	BP	0,000296075
GO:0009991	48	197	response to extracellular stimulus	BP	0,000300193
GO:0031327	49	211	negative regulation of cellular biosynthetic process	BP	0,000300629
GO:0005839	7	8	proteasome core complex	CC	0,000301899
GO:0010499	7	8	proteasomal ubiquitin-independent protein catabolic process	BP	0,000301899
GO:0015980	17	45	energy derivation by oxidation of organic compounds	BP	0,000306458
GO:0051276	46	219	chromosome organization	BP	0,000314562
GO:0000147	6	6	actin cortical patch assembly	BP	0,00031833
GO:0030866	6	6	cortical actin cytoskeleton organization	BP	0,00031833
GO:0034314	6	6	Arp2/3 complex-mediated actin nucleation	BP	0,00031833
GO:0071248	18	46	cellular response to metal ion	BP	0,000324751

GO:0009267	31	110	cellular response to starvation	BP	0,000342686
GO:0008064	9	15	regulation of actin polymerization or depolymerization	BP	0,000357073
GO:0030832	9	15	regulation of actin filament length	BP	0,000357073
GO:0032786	5	5	positive regulation of DNA-templated transcription, elongation	BP	0,00036419
GO:0072686	12	25	mitotic spindle	CC	0,000370784
GO:0009272	8	11	fungal-type cell wall biogenesis	BP	0,000382598
GO:0005198	59	224	structural molecule activity	MF	0,000386231
GO:0005856	60	270	cytoskeleton	CC	0,000390023
GO:0030004	10	18	cellular monovalent inorganic cation homeostasis	BP	0,000403057
GO:0032271	9	15	regulation of protein polymerization	BP	0,00040595
GO:0090066	16	40	regulation of anatomical structure size	BP	0,000418886
GO:0016282	5	5	eukaryotic 43S preinitiation complex	CC	0,00042901
GO:0070993	5	5	translation preinitiation complex	CC	0,00042901
GO:0032879	30	104	regulation of localization	BP	0,000437869
GO:0042790	4	4	nucleolar large rRNA transcription by RNA polymerase I	BP	0,000449643

GO:0045898	6	7	regulation of RNA polymerase II transcriptional preinitiation complex assembly	BP	0,000456875
GO:0031334	9	16	positive regulation of protein complex assembly	BP	0,000470263
GO:0006446	9	14	regulation of translational initiation	BP	0,000474691
GO:0043248	8	11	proteasome assembly	BP	0,000495032
GO:0005643	10	21	nuclear pore	CC	0,000497079
GO:0006605	39	156	protein targeting	BP	0,000535308
GO:0008540	6	7	proteasome regulatory particle, base subcomplex	CC	0,000563782
GO:0012507	5	5	ER to Golgi transport vesicle membrane	CC	0,000563782
GO:0006184	20	55	NA	NA	0,000571911
GO:1901069	20	55	guanosine-containing compound catabolic process	BP	0,000571911
GO:0018130	63	280	heterocycle biosynthetic process	BP	0,000572433
GO:0034728	7	10	nucleosome organization	BP	0,000572433
GO:0044272	21	58	sulfur compound biosynthetic process	BP	0,000580803
GO:0065009	53	224	regulation of molecular function	BP	0,000658323
GO:0006399	19	56	tRNA metabolic process	BP	0,000659584

GO:0001411	5	5	hyphal tip	CC	0,00066146
GO:0022406	7	10	membrane docking	BP	0,00066146
GO:0048278	7	10	vesicle docking	BP	0,00066146
GO:0045324	8	13	late endosome to vacuole transport	BP	0,000663133
GO:0000463	8	12	maturation of LSU-rRNA from tricistronic rRNA transcript (SSU-rRNA, 5.8S rRNA, LSU-rRNA)	BP	0,000689838
GO:0010638	13	33	positive regulation of organelle organization	BP	0,000690305
GO:0006417	23	70	regulation of translation	BP	0,000748515
GO:0046039	20	56	GTP metabolic process	BP	0,000779414
GO:0043614	5	5	multi-eIF complex	CC	0,000781025
GO:0007584	12	26	response to nutrient	BP	0,000784345
GO:0043044	6	9	ATP-dependent chromatin remodeling	BP	0,000790231
GO:0000966	9	18	RNA 5'-end processing	BP	0,000790231
GO:0045899	5	5	positive regulation of RNA polymerase II transcriptional preinitiation complex assembly	BP	0,000804657
GO:0030234	34	122	enzyme regulator activity	MF	0,000883869
GO:0009060	11	23	aerobic respiration	BP	0,000883869

GO:0051253	42	179	negative regulation of RNA metabolic process	BP	0,000898898
GO:0051129	13	30	negative regulation of cellular component organization	BP	0,000915729
GO:0046930	10	22	pore complex	CC	0,000933893
GO:0009404	13	26	toxin metabolic process	BP	0,000967241
GO:0010970	6	7	transport along microtubule	BP	0,000971242
GO:0005975	64	300	carbohydrate metabolic process	BP	0,000971242
GO:0035556	34	120	intracellular signal transduction	BP	0,000975364
GO:0048583	74	354	regulation of response to stimulus	BP	0,000981067
GO:0030674	6	8	protein binding, bridging	MF	0,000999651
GO:0032268	41	164	regulation of cellular protein metabolic process	BP	0,001032362
GO:0072524	15	35	pyridine-containing compound metabolic process	BP	0,001061084
GO:0007530	4	4	sex determination	BP	0,001061601
GO:0007531	4	4	mating type determination	BP	0,001061601
GO:0031670	7	11	cellular response to nutrient	BP	0,001113275
GO:0044282	33	121	small molecule catabolic process	BP	0,001113275

GO:0044712	33	121	NA	NA	0,001113275
GO:0043648	18	50	dicarboxylic acid metabolic process	BP	0,00111646
GO:0000447	10	19	endonucleolytic cleavage in ITS1 to separate SSU-rRNA from 5.8S rRNA and LSU-rRNA from tricistronic rRNA transcript (SSU-rRNA, 5.8S rRNA, LSU-rRNA)	BP	0,001152914
GO:0000478	10	19	endonucleolytic cleavage involved in rRNA processing	BP	0,001152914
GO:0000479	10	19	endonucleolytic cleavage of tricistronic rRNA transcript (SSU-rRNA, 5.8S rRNA, LSU-rRNA)	BP	0,001152914
GO:0090502	10	19	RNA phosphodiester bond hydrolysis, endonucleolytic	BP	0,001152914
GO:0006790	35	127	sulfur compound metabolic process	BP	0,001201512
GO:0048522	82	399	positive regulation of cellular process	BP	0,001235708
GO:0009890	49	221	negative regulation of biosynthetic process	BP	0,001261117
GO:0000785	20	66	chromatin	CC	0,001263738
GO:0031324	55	262	negative regulation of cellular metabolic process	BP	0,001266602
GO:1901068	20	58	guanosine-containing compound metabolic process	BP	0,001338413
GO:1901988	17	43	negative regulation of cell cycle phase transition	BP	0,001361188
GO:0008139	3	3	nuclear localization sequence binding	MF	0,001380846
GO:0016197	18	50	endosomal transport	BP	0,001380846

GO:0051235	9	18	maintenance of location	BP	0,001380846
GO:0000915	5	5	actomyosin contractile ring assembly	BP	0,001388376
GO:0019773	6	7	proteasome core complex, alpha-subunit complex	CC	0,001405173
GO:0060590	6	7	ATPase regulator activity	MF	0,001424465
GO:0051345	16	41	positive regulation of hydrolase activity	BP	0,001424986
GO:0016054	30	107	organic acid catabolic process	BP	0,001461326
GO:0046395	30	107	carboxylic acid catabolic process	BP	0,001461326
GO:0030833	8	14	regulation of actin filament polymerization	BP	0,001461326
GO:0003924	19	54	GTPase activity	MF	0,001466401
GO:0044732	11	24	mitotic spindle pole body	CC	0,001488651
GO:0006099	8	13	tricarboxylic acid cycle	BP	0,001490788
GO:0044430	41	176	cytoskeletal part	CC	0,00153461
GO:0008135	14	36	translation factor activity, RNA binding	MF	0,001535215
GO:0009112	10	20	nucleobase metabolic process	BP	0,001564382
GO:0030473	5	5	nuclear migration along microtubule	BP	0,001584467

GO:0003729	19	58	mRNA binding	MF	0,001621241
GO:0042147	6	7	retrograde transport, endosome to Golgi	BP	0,001621241
GO:0009814	44	184	defense response, incompatible interaction	BP	0,001636727
GO:0031123	12	32	RNA 3'-end processing	BP	0,001640345
GO:0031410	32	128	cytoplasmic vesicle	CC	0,001647796
GO:0031966	48	192	mitochondrial membrane	CC	0,001674915
GO:1901991	14	32	negative regulation of mitotic cell cycle phase transition	BP	0,001695125
GO:0045185	8	15	maintenance of protein location	BP	0,001747423
GO:0008134	5	8	transcription factor binding	MF	0,001758869
GO:0003688	6	8	DNA replication origin binding	MF	0,001813435
GO:0042026	6	8	protein refolding	BP	0,001820799
GO:0030863	11	27	cortical cytoskeleton	CC	0,001895881
GO:0051301	32	132	cell division	BP	0,001895881
GO:0000746	9	15	conjugation	BP	0,001910192
GO:0000747	9	15	conjugation with cellular fusion	BP	0,001910192

GO:0001076	3	3	NA	NA	0,001932388
GO:0000910	21	72	cytokinesis	BP	0,002019057
GO:0007154	132	756	cell communication	BP	0,002046772
GO:0016860	16	40	intramolecular oxidoreductase activity	MF	0,002082439
GO:0000387	4	4	spliceosomal snRNP assembly	BP	0,002100882
GO:0003743	11	26	translation initiation factor activity	MF	0,002167838
GO:0072350	8	13	tricarboxylic acid metabolic process	BP	0,002186299
GO:0006354	5	6	DNA-templated transcription, elongation	BP	0,002233841
GO:0031126	5	6	snoRNA 3'-end processing	BP	0,002294348
GO:0070887	121	685	cellular response to chemical stimulus	BP	0,002387472
GO:0032273	7	12	positive regulation of protein polymerization	BP	0,002430129
GO:0005783	113	589	endoplasmic reticulum	CC	0,002448081
GO:0060090	6	10	molecular adaptor activity	MF	0,002450679
GO:0005852	7	12	eukaryotic translation initiation factor 3 complex	CC	0,002460477
GO:0005789	54	240	endoplasmic reticulum membrane	CC	0,002489659

GO:0031000	4	4	response to caffeine	BP	0,002489659
GO:0043279	4	4	response to alkaloid	BP	0,002489659
GO:0071312	4	4	cellular response to alkaloid	BP	0,002489659
GO:0071313	4	4	cellular response to caffeine	BP	0,002489659
GO:0071415	4	4	cellular response to purine-containing compound	BP	0,002489659
GO:0014074	5	7	response to purine-containing compound	BP	0,002498887
GO:0030118	5	7	clathrin coat	CC	0,0025102
GO:0030125	5	7	clathrin vesicle coat	CC	0,0025102
GO:0044723	53	238	NA	NA	0,002585321
GO:0051172	44	202	negative regulation of nitrogen compound metabolic process	BP	0,002585321
GO:0006970	95	475	response to osmotic stress	BP	0,002586325
GO:0045892	40	177	negative regulation of transcription, DNA-templated	BP	0,002604875
GO:0019843	14	33	rRNA binding	MF	0,002628464
GO:0006012	11	22	galactose metabolic process	BP	0,002653917
GO:0009303	4	6	rRNA transcription	BP	0,002676925

GO:0030127	4	4	COPII vesicle coat	CC	0,002715281
GO:0006914	11	25	autophagy	BP	0,002827303
GO:0065004	8	16	protein-DNA complex assembly	BP	0,002858028
GO:0042176	7	11	regulation of protein catabolic process	BP	0,002878935
GO:0051495	8	15	positive regulation of cytoskeleton organization	BP	0,002886095
GO:0044444	927	6482	cytoplasmic part	CC	0,002969497
GO:0031328	45	194	positive regulation of cellular biosynthetic process	BP	0,003001151
GO:0051050	10	21	positive regulation of transport	BP	0,003002693
GO:0004004	8	14	ATP-dependent RNA helicase activity	MF	0,003043793
GO:0030705	6	8	cytoskeleton-dependent intracellular transport	BP	0,003097824
GO:0019897	8	14	extrinsic component of plasma membrane	CC	0,003105152
GO:0042594	32	131	response to starvation	BP	0,003151769
GO:0051346	6	8	negative regulation of hydrolase activity	BP	0,003219009
GO:0009066	17	49	aspartate family amino acid metabolic process	BP	0,003312749
GO:0007035	7	11	vacuolar acidification	BP	0,003312749

GO:0045851	7	11	pH reduction	BP	0,003312749
GO:0051452	7	11	intracellular pH reduction	BP	0,003312749
GO:1901698	47	212	response to nitrogen compound	BP	0,003312749
GO:0006323	8	18	DNA packaging	BP	0,003328659
GO:0051170	11	29	import into nucleus	BP	0,003328659
GO:0010558	44	203	negative regulation of macromolecule biosynthetic process	BP	0,003368031
GO:2000113	44	203	negative regulation of cellular macromolecule biosynthetic process	BP	0,003368031
GO:0009891	45	195	positive regulation of biosynthetic process	BP	0,003368031
GO:0006749	10	21	glutathione metabolic process	BP	0,003455703
GO:0071218	4	4	cellular response to misfolded protein	BP	0,003477833
GO:0044427	34	155	chromosomal part	CC	0,003482798
GO:0032506	15	44	cytokinetic process	BP	0,003482798
GO:0000049	3	3	tRNA binding	MF	0,003496405
GO:0006885	8	15	regulation of pH	BP	0,003504172
GO:0071013	6	8	catalytic step 2 spliceosome	CC	0,003511904

GO:0016471	6	8	vacuolar proton-transporting V-type ATPase complex	CC	0,003511904
GO:0033176	6	8	proton-transporting V-type ATPase complex	CC	0,003511904
GO:0048518	97	511	positive regulation of biological process	BP	0,003605391
GO:0010639	10	23	negative regulation of organelle organization	BP	0,003705698
GO:0051049	26	97	regulation of transport	BP	0,003707344
GO:0045934	43	200	negative regulation of nucleobase-containing compound metabolic process	BP	0,003720119
GO:0046822	5	6	regulation of nucleocytoplasmic transport	BP	0,003800156
GO:0030658	8	16	transport vesicle membrane	CC	0,003837496
GO:0006356	4	4	regulation of transcription by RNA polymerase I	BP	0,003837496
GO:0006283	4	4	transcription-coupled nucleotide-excision repair	BP	0,003837496
GO:0005682	5	6	U5 snRNP	CC	0,004003499
GO:0060589	17	53	nucleoside-triphosphatase regulator activity	MF	0,00403775
GO:0035967	8	14	cellular response to topologically incorrect protein	BP	0,00403775
GO:0032968	4	4	positive regulation of transcription elongation from RNA polymerase II promoter	BP	0,00404257
GO:0019438	72	349	aromatic compound biosynthetic process	BP	0,004045766

GO:0009067	12	29	aspartate family amino acid biosynthetic process	BP	0,004065912
GO:0055114	108	552	oxidation-reduction process	BP	0,004074339
GO:0048284	13	33	organelle fusion	BP	0,004143378
GO:0072665	13	36	protein localization to vacuole	BP	0,004242814
GO:0051258	6	9	protein polymerization	BP	0,004307464
GO:0005759	26	89	mitochondrial matrix	CC	0,004334593
GO:0006376	6	8	mRNA splice site selection	BP	0,004448965
GO:0009898	8	15	cytoplasmic side of plasma membrane	CC	0,004478107
GO:0070647	46	211	protein modification by small protein conjugation or removal	BP	0,004478107
GO:0030641	7	12	regulation of cellular pH	BP	0,004478107
GO:0051453	7	12	regulation of intracellular pH	BP	0,004478107
GO:0031982	34	146	vesicle	CC	0,004691081
GO:0004843	6	10	thiol-dependent ubiquitin-specific protease activity	MF	0,004799601
GO:0000790	15	46	nuclear chromatin	CC	0,004799601
GO:0019898	15	44	extrinsic component of membrane	CC	0,004879439

GO:0048610	52	258	NA	NA	0,004932337
GO:0000220	5	6	vacuolar proton-transporting V-type ATPase, V0 domain	CC	0,005014283
GO:0033179	5	6	proton-transporting V-type ATPase, V0 domain	CC	0,005014283
GO:1901987	18	52	regulation of cell cycle phase transition	BP	0,005109355
GO:0042221	324	2025	response to chemical	BP	0,005144481
GO:0000470	8	15	maturation of LSU-rRNA	BP	0,005266861
GO:0031234	7	12	extrinsic component of cytoplasmic side of plasma membrane	CC	0,00532855
GO:0034976	8	14	response to endoplasmic reticulum stress	BP	0,005424899
GO:0045935	40	174	positive regulation of nucleobase-containing compound metabolic process	BP	0,005444694
GO:0017069	4	5	snRNA binding	MF	0,005444694
GO:0008186	8	15	RNA-dependent ATPase activity	MF	0,005444694
GO:0004364	10	20	glutathione transferase activity	MF	0,005595705
GO:0005686	6	9	U2 snRNP	CC	0,005719732
GO:0080135	12	33	regulation of cellular response to stress	BP	0,005963717
GO:0044454	21	81	nuclear chromosome part	CC	0,005964328

GO:0030544	5	6	Hsp70 protein binding	MF	0,006006544
GO:0006334	4	5	nucleosome assembly	BP	0,006062597
GO:0006094	5	6	gluconeogenesis	BP	0,006085714
GO:0060416	7	11	response to growth hormone	BP	0,006107191
GO:0048280	4	4	vesicle fusion with Golgi apparatus	BP	0,006325026
GO:0005484	8	14	SNAP receptor activity	MF	0,006450201
GO:0055067	11	28	monovalent inorganic cation homeostasis	BP	0,006649415
GO:0001671	5	6	ATPase activator activity	MF	0,006696196
GO:0032781	5	6	positive regulation of ATPase activity	BP	0,006696196
GO:0043462	5	6	regulation of ATPase activity	BP	0,006696196
GO:0080090	209	1265	regulation of primary metabolic process	BP	0,00672631
GO:0071705	47	208	nitrogen compound transport	BP	0,006772304
GO:0009298	4	4	GDP-mannose biosynthetic process	BP	0,006772304
GO:0019673	4	4	GDP-mannose metabolic process	BP	0,006772304
GO:0000395	5	6	mRNA 5'-splice site recognition	BP	0,006890257

GO:0061135	5	6	endopeptidase regulator activity	MF	0,006953954
GO:0006413	18	57	translational initiation	BP	0,007082751
GO:0051285	4	4	cell cortex of cell tip	CC	0,00713834
GO:0000122	12	31	negative regulation of transcription by RNA polymerase II	BP	0,007162371
GO:0000075	15	41	cell cycle checkpoint	BP	0,007243044
GO:0016074	5	7	snoRNA metabolic process	BP	0,00735583
GO:0043144	5	7	snoRNA processing	BP	0,00735583
GO:0065008	89	485	regulation of biological quality	BP	0,007375473
GO:0005732	6	9	small nucleolar ribonucleoprotein complex	CC	0,007400424
GO:0033124	12	31	NA	NA	0,007500825
GO:0043087	12	31	regulation of GTPase activity	BP	0,007500825
GO:0006464	125	746	cellular protein modification process	BP	0,007538025
GO:0036211	125	746	protein modification process	BP	0,007538025
GO:0006606	10	27	protein import into nucleus	BP	0,007593902
GO:0044744	10	27	NA	NA	0,007593902

GO:0042995	16	48	cell projection	CC	0,007787852
GO:0051173	41	182	positive regulation of nitrogen compound metabolic process	BP	0,007800172
GO:0008023	4	5	transcription elongation factor complex	CC	0,007838222
GO:0016469	9	18	proton-transporting two-sector ATPase complex	CC	0,007838222
GO:0031338	4	5	regulation of vesicle fusion	BP	0,007919565
GO:0035542	4	5	regulation of SNARE complex assembly	BP	0,007919565
GO:0070682	4	4	proteasome regulatory particle assembly	BP	0,008085737
GO:0070127	4	5	tRNA aminoacylation for mitochondrial protein translation	BP	0,008102163
GO:0008843	4	4	endochitinase activity	MF	0,008179128
GO:0030838	6	11	positive regulation of actin filament polymerization	BP	0,008181739
GO:0045010	6	11	actin nucleation	BP	0,008181739
GO:0030702	4	5	chromatin silencing at centromere	BP	0,008216072
GO:0009407	10	21	toxin catabolic process	BP	0,008549361
GO:0030003	16	54	cellular cation homeostasis	BP	0,008565696
GO:0010557	42	188	positive regulation of macromolecule biosynthetic process	BP	0,008584008

GO:0007064	4	5	mitotic sister chromatid cohesion	BP	0,008633495
GO:0006515	4	5	protein quality control for misfolded or incompletely synthesized proteins	BP	0,008648718
GO:0031981	137	782	nuclear lumen	CC	0,008883139
GO:0030659	21	82	cytoplasmic vesicle membrane	CC	0,009084994
GO:0005782	6	10	peroxisomal matrix	CC	0,009084994
GO:0031907	6	10	microbody lumen	CC	0,009084994
GO:0030897	3	3	HOPS complex	CC	0,009084994
GO:0033263	3	3	CORVET complex	CC	0,009084994
GO:0019200	8	18	carbohydrate kinase activity	MF	0,009115883
GO:0006342	15	52	chromatin silencing	BP	0,009325238
GO:0045814	15	52	negative regulation of gene expression, epigenetic	BP	0,009325238
GO:0006368	4	5	transcription elongation from RNA polymerase II promoter	BP	0,00933951
GO:0034504	12	36	protein localization to nucleus	BP	0,009471101
GO:0030952	5	7	establishment or maintenance of cytoskeleton polarity	BP	0,009500861
GO:0006896	6	10	Golgi to vacuole transport	BP	0,00950443

GO:0008443	5	7	phosphofructokinase activity	MF	0,009744003
GO:0045013	4	5	carbon catabolite repression of transcription	BP	0,009789888
GO:0045014	4	5	carbon catabolite repression of transcription by glucose	BP	0,009789888
GO:0046015	4	5	regulation of transcription by glucose	BP	0,009789888
GO:0005885	6	9	Arp2/3 protein complex	CC	0,009966764
CLUSTER 3					
GO:0009962	19	44	regulation of flavonoid biosynthetic process	BP	1,17E-15
GO:0009813	21	59	flavonoid biosynthetic process	BP	2,80E-15
GO:0009812	21	63	flavonoid metabolic process	BP	4,63E-15
GO:0031540	16	31	regulation of anthocyanin biosynthetic process	BP	1,25E-14
GO:0031537	16	40	regulation of anthocyanin metabolic process	BP	3,37E-13
GO:0009914	18	83	hormone transport	BP	1,10E-11
GO:0009926	17	78	auxin polar transport	BP	3,84E-11
GO:0005774	47	591	vacuolar membrane	CC	3,84E-11
GO:0060918	17	80	auxin transport	BP	4,05E-11
GO:004443	47	595	vacuolar part	CC	4,05E-11

7					
GO:0009629	16	68	response to gravity	BP	3,73E-10
GO:0010224	19	95	response to UV-B	BP	7,91E-10
GO:0009705	18	92	plant-type vacuole membrane	CC	4,30E-09
GO:0000325	19	116	plant-type vacuole	CC	8,99E-09
GO:0009411	19	122	response to UV	BP	2,48E-08
GO:0010817	19	142	regulation of hormone levels	BP	4,64E-08
GO:0005773	53	920	vacuole	CC	9,55E-08
GO:0009753	22	203	response to jasmonic acid	BP	3,23E-07
GO:0006979	29	344	response to oxidative stress	BP	5,71E-07
GO:0009611	23	236	response to wounding	BP	2,40E-06
GO:0031090	65	1448	organelle membrane	CC	4,43E-06
GO:0022626	29	336	cytosolic ribosome	CC	7,15E-06
GO:0044391	26	273	ribosomal subunit	CC	7,80E-06
GO:0044445	29	361	cytosolic part	CC	2,60E-05
GO:000574	10	43	mitochondrial respiratory chain	CC	3,23E-05

6					
GO:0005840	29	363	ribosome	CC	3,23E-05
GO:0070469	10	44	respiratory chain	CC	3,86E-05
GO:0009733	20	234	response to auxin	BP	4,95E-05
GO:0009416	30	521	response to light stimulus	BP	0,001064035
GO:0005747	7	27	mitochondrial respiratory chain complex I	CC	0,001241434
GO:0030964	7	28	NADH dehydrogenase complex	CC	0,001535124
GO:0045271	7	28	respiratory chain complex I	CC	0,001535124
GO:0009314	30	545	response to radiation	BP	0,001930721
GO:0044765	40	901	NA	NA	0,002294764
GO:0030529	29	479	NA	NA	0,002455286
GO:0044455	10	77	mitochondrial membrane part	CC	0,004823635
GO:0022625	14	146	cytosolic large ribosomal subunit	CC	0,009477838
CLUSTER 5					
GO:0010497	3	9	plasmodesmata-mediated intercellular transport	BP	0,000552099
GO:0010496	3	11	intercellular transport	BP	0,000552099

GO:2000280	3	18	regulation of root development	BP	0,00153118
CLUSTER 11					
GO:0045471	5	25	response to ethanol	BP	0,000186179
GO:0046685	5	40	response to arsenic-containing substance	BP	0,00079021
GO:0046688	5	50	response to copper ion	BP	0,001198464
GO:0009408	7	219	response to heat	BP	0,004904004
CLUSTER 18					
GO:0006259	32	306	DNA metabolic process	BP	0,002659186
GO:0015098	4	5	molybdate ion transmembrane transporter activity	MF	0,007142342
GO:0015689	4	5	molybdate ion transport	BP	0,007142342
CLUSTER 19					
GO:0009507	251	2519	chloroplast	CC	1,30E-21
GO:0009536	254	2607	plastid	CC	2,04E-21
GO:0010588	76	246	cotyledon vascular tissue pattern formation	BP	8,52E-17
GO:0010087	79	274	phloem or xylem histogenesis	BP	1,76E-16
GO:0010305	76	271	leaf vascular tissue pattern formation	BP	3,36E-15
GO:001005	78	296	xylem and phloem pattern formation	BP	9,71E-15

1					
GO:0003002	84	360	regionalization	BP	6,25E-14
GO:0007389	87	385	pattern specification process	BP	6,25E-14
GO:0048827	100	563	phyllome development	BP	5,55E-13
GO:0048731	172	1476	system development	BP	1,32E-12
GO:0048367	110	686	shoot system development	BP	1,62E-12
GO:0048366	89	468	leaf development	BP	2,50E-12
GO:0009888	105	682	tissue development	BP	3,04E-11
GO:0044707	201	2047	NA	NA	7,24E-11
GO:0032501	202	2089	multicellular organismal process	BP	1,69E-10
GO:0007275	194	1982	multicellular organism development	BP	4,44E-10
GO:0044767	170	1716	NA	NA	2,90E-09
GO:0048856	189	2002	anatomical structure development	BP	6,54E-09
GO:0032502	197	2182	developmental process	BP	4,66E-08
GO:0044699	291	4159	NA	NA	0,000195
GO:000573	411	7138	cytoplasm	CC	0,003264673

7					
GO:0044444	382	6482	cytoplasmic part	CC	0,004541134
GO:0010154	48	379	fruit development	BP	0,004697074
GO:0009791	83	868	post-embryonic development	BP	0,00855277
GO:0048316	46	366	seed development	BP	0,00883263
GO:0048608	69	663	reproductive structure development	BP	0,00883263
GO:0061458	69	663	reproductive system development	BP	0,00883263
CLUSTER 24					
GO:0046658	11	58	anchored component of plasma membrane	CC	0,001061202
GO:0071944	96	2502	cell periphery	CC	0,00342024
GO:0031226	13	113	intrinsic component of plasma membrane	CC	0,008492074
CLUSTER 27					
GO:0006720	8	108	isoprenoid metabolic process	BP	0,00140326
GO:0009507	33	2519	chloroplast	CC	0,00140326
GO:0016108	5	28	tetraterpenoid metabolic process	BP	0,00140326
GO:0016116	5	28	carotenoid metabolic process	BP	0,00140326
GO:000672	7	84	terpenoid metabolic process	BP	0,00140326

1					
GO:0008299	7	88	isoprenoid biosynthetic process	BP	0,001575028
GO:0009536	33	2607	plastid	CC	0,001575028
GO:0016114	6	64	terpenoid biosynthetic process	BP	0,003031745
GO:0016109	4	20	tetraterpenoid biosynthetic process	BP	0,005398551
GO:0016117	4	20	carotenoid biosynthetic process	BP	0,005398551
CLUSTER 29					
GO:0016328	5	11	lateral plasma membrane	CC	0,002907977
GO:0048766	4	5	root hair initiation	BP	0,002907977
