# Supplementary material for: The transcriptome of Pinus pinaster under Fusarium circinatum challenge
Source: BMC Genomics. 2020 Jan 8;21:28. doi: 10.1186/s12864-019-6444-0 (PMC6950806; doi:10.1186/s12864-019-6444-0)
Supplement: Supplementary file 13 — Additional file 13: Significantly enriched GO terms identified from high confidence expressed Fusarium circinatum genes. [file 12864_2019_6444_MOESM13_ESM.pdf]

Additional file 13: Significantly enriched GO terms identified from high confidence expressed *Fusarium circinatum* genes.

category	numDEInCat	numInCat	term	ontology	FDR overrepresented p-value
CLUSTER 3					
GO:0005737	1863	3526	cytoplasm	CC	1,45E-24
GO:0044429	319	457	mitochondrial part	CC	2,91E-24
GO:0044444	1696	3174	cytoplasmic part	CC	1,48E-22
GO:0003735	94	115	structural constituent of ribosome	MF	8,40E-16
GO:0031090	578	943	organelle membrane	CC	8,40E-16
GO:0044391	100	125	ribosomal subunit	CC	3,03E-15
GO:0005575	1985	3849	cellular_component	CC	1,24E-14
GO:0005622	2066	4070	intracellular	CC	1,31E-13
GO:0019866	137	183	organelle inner membrane	CC	2,37E-13
GO:0005740	210	309	mitochondrial envelope	CC	2,51E-13
GO:0031966	191	275	mitochondrial membrane	CC	2,99E-13
GO:0005743	131	175	mitochondrial inner membrane	CC	4,21E-13
GO:0005739	552	935	mitochondrion	CC	5,55E-13
GO:0044424	2051	4041	intracellular part	CC	9,75E-13
GO:0005840	115	158	ribosome	CC	8,26E-12
GO:0005623	2098	4168	cell	CC	7,75E-11
GO:0044464	2098	4168	cell part	CC	7,75E-11
GO:0031967	291	458	organelle envelope	CC	8,26E-11
GO:0031975	291	458	envelope	CC	8,26E-11
GO:0015934	59	70	large ribosomal subunit	CC	8,81E-11
GO:0043229	1886	3692	intracellular organelle	CC	1,10E-10
GO:0043226	1886	3693	organelle	CC	1,30E-10
GO:0005759	129	180	mitochondrial matrix	CC	1,40E-10

GO:0005761	44	48	mitochondrial ribosome	CC	5,79E-10
GO:0007005	154	228	mitochondrion organization	BP	6,30E-09
GO:0044455	83	108	mitochondrial membrane part	CC	6,68E-09
GO:0005198	131	193	structural molecule activity	MF	1,63E-08
GO:0043231	1816	3565	intracellular membrane-bounded organelle	CC	2,25E-08
GO:0043227	1816	3566	membrane-bounded organelle	CC	2,60E-08
GO:0000313	36	39	organellar ribosome	CC	3,88E-08
GO:0016020	819	1473	membrane	CC	5,35E-08
GO:0044446	1214	2289	intracellular organelle part	CC	3,20E-07
GO:0044422	1215	2292	organelle part	CC	3,48E-07
GO:0003674	1517	2908	molecular_function	MF	3,80E-07
GO:0046907	346	577	intracellular transport	BP	1,86E-06
GO:0044425	544	955	membrane part	CC	2,16E-06
GO:0006839	66	88	mitochondrial transport	BP	2,50E-06
GO:0035091	48	55	phosphatidylinositol binding	MF	3,54E-06
GO:0051649	369	623	establishment of localization in cell	BP	6,88E-06
GO:0006091	84	117	generation of precursor metabolites and energy	BP	6,88E-06
GO:1901981	38	42	phosphatidylinositol phosphate binding	MF	1,22E-05
GO:0009987	1901	3768	cellular process	BP	1,84E-05
GO:0055114	238	406	oxidation-reduction process	BP	2,04E-05
GO:0000314	19	19	organellar small ribosomal subunit	CC	2,84E-05
GO:0005763	19	19	mitochondrial small ribosomal subunit	CC	2,84E-05
GO:0022626	60	86	cytosolic ribosome	CC	4,50E-05
GO:0022625	33	41	cytosolic large ribosomal subunit	CC	4,56E-05
GO:0072594	120	179	establishment of protein localization to organelle	BP	4,79E-05
GO:0000315	25	28	organellar large ribosomal subunit	CC	7,32E-05
GO:0005762	25	28	mitochondrial large ribosomal subunit	CC	7,32E-05

GO:0044432	220	363	endoplasmic reticulum part	CC	8,99E-05
GO:0045184	213	348	establishment of protein localization	BP	0,00011661
GO:0006412	152	253	translation	BP	0,000125333
GO:0015935	42	56	small ribosomal subunit	CC	0,000132753
GO:0006605	136	208	protein targeting	BP	0,000132753
GO:0045333	50	67	cellular respiration	BP	0,000186696
GO:0051641	448	784	cellular localization	BP	0,000190806
GO:0006886	200	327	intracellular protein transport	BP	0,000230395
GO:0005543	54	68	phospholipid binding	MF	0,00023254
GO:0031300	94	140	intrinsic component of organelle membrane	CC	0,000251802
GO:0044283	181	303	small molecule biosynthetic process	BP	0,000257735
GO:0005783	311	538	endoplasmic reticulum	CC	0,000298382
GO:0006119	19	20	oxidative phosphorylation	BP	0,000298382
GO:0009060	41	53	aerobic respiration	BP	0,000347756
GO:0015031	203	333	protein transport	BP	0,000347756
GO:0065002	51	69	intracellular protein transmembrane transport	BP	0,000347756
GO:0071806	51	69	protein transmembrane transport	BP	0,000347756
GO:0008289	65	88	lipid binding	MF	0,000568027
GO:0016482	256	432	cytosolic transport	BP	0,000568027
GO:0031301	90	136	integral component of organelle membrane	CC	0,00082487
GO:0044743	44	59	protein transmembrane import into intracellular organelle	BP	0,000922253
GO:0070585	41	55	protein localization to mitochondrion	BP	0,001097827
GO:0072655	41	55	establishment of protein localization to mitochondrion	BP	0,001097827
GO:0044267	570	1059	cellular protein metabolic process	BP	0,001135108
GO:0015980	64	92	energy derivation by oxidation of organic compounds	BP	0,001211081
GO:0044445	92	148	cytosolic part	CC	0,001863261
GO:0019538	586	1093	protein metabolic process	BP	0,002006641

GO:0032543	57	84	mitochondrial translation	BP	0,002402531
GO:0005789	201	340	endoplasmic reticulum membrane	CC	0,002521265
GO:0005794	191	317	Golgi apparatus	CC	0,002550504
GO:0006626	39	53	protein targeting to mitochondrion	BP	0,002640899
GO:0032266	19	20	phosphatidylinositol-3-phosphate binding	MF	0,002787522
GO:0042773	15	16	ATP synthesis coupled electron transport	BP	0,00373535
GO:0042775	15	16	mitochondrial ATP synthesis coupled electron transport	BP	0,00373535
GO:1901566	206	360	organonitrogen compound biosynthetic process	BP	0,00380941
GO:0005746	19	22	mitochondrial respiratory chain	CC	0,00499503
GO:0070469	19	22	respiratory chain	CC	0,00499503
GO:1901576	674	1282	organic substance biosynthetic process	BP	0,005002191
GO:0006629	172	288	lipid metabolic process	BP	0,005590454
GO:0006810	517	939	transport	BP	0,005590454
GO:0017038	61	88	protein import	BP	0,005983178
GO:0006007	24	29	glucose catabolic process	BP	0,006165119
GO:0006733	43	60	oxidoreduction coenzyme metabolic process	BP	0,006165119
GO:0031970	36	50	organelle envelope lumen	CC	0,006165119
GO:0019725	101	159	cellular homeostasis	BP	0,007574157
GO:0044765	455	821	NA	NA	0,007949213
GO:0042175	203	350	nuclear outer membrane-endoplasmic reticulum membrane network	CC	0,008473093
GO:0007007	14	15	inner mitochondrial membrane organization	BP	0,009352847
CLUSTER 4					
GO:0007165	37	311	signal transduction	BP	8,88E-05
GO:0023052	37	322	signaling	BP	8,88E-05
GO:0044700	37	322	NA	NA	8,88E-05
GO:0050794	89	1306	regulation of cellular process	BP	0,000216086
GO:0050789	92	1374	regulation of biological process	BP	0,000216086

GO:0065007	98	1545	biological regulation	BP	0,000595766
GO:0048522	40	462	positive regulation of cellular process	BP	0,003951215
GO:0000079	6	14	regulation of cyclin-dependent protein serine/threonine kinase activity	BP	0,003951215
GO:0005034	3	5	osmosensor activity	MF	0,004464935
GO:0048518	41	486	positive regulation of biological process	BP	0,0051923
GO:0030427	26	175	site of polarized growth	CC	0,00570653
GO:0016538	5	18	cyclin-dependent protein serine/threonine kinase regulator activity	MF	0,00692769
