# Supplementary material for: The transcriptome of Pinus pinaster under Fusarium circinatum challenge
Source: BMC Genomics. 2020 Jan 8;21:28. doi: 10.1186/s12864-019-6444-0 (PMC6950806; doi:10.1186/s12864-019-6444-0)
Supplement: Supplementary file 14 — Additional file 14: Hormone related differential expressed (DE) genes in Fusarium circinatum. [file 12864_2019_6444_MOESM14_ESM.pdf]

Additional file 14: Hormone related differential expressed (DE) genes in *Fusarium circinatum*.

	Log2 (Fold Change) value							
Sequence ID	3v5 dpi	Corrected p-value	3v10 dpi	Corrected p-value	5v10 dpi	Corrected p-value	Predicted Gene	Description
Enzymes-related to GA biosynthesis								
FCIRG_05400T1	-6,4482	9,98E-09	-8,5149	1.87E-06	0,0000			Gibberellin cluster-kauren synthase
FCIRG_05399T1	-7,1855	6,73E-06	-9,6374	1.45E-13	-2,4519	0.003006		Gibberellin cluster-C13-oxidase
FCIRG_05404T1	-5,5082	1,03E-05	-7,1938	1.01E-12	0,0000			Gibberellin cluster-GA14-synthase
FCIRG_05401T1	-6,7532	3,02E-02	-9,1462	3.77E-17	-2,3930	0.030750		Gibberellin cluster-GGPP-synthase
Enzymes-related to Ethylene biosynthesis								
FCIRG_14291T1	-4,0340	0.000181	-7,6199	1.09E-12	-3,5859	6.82E-05		2-oxoglutarate-dependent ethylene succinate-forming enzyme
FCIRG_01209T1	0,0000		-2,6599	9.14E-05	-1,7377	0.023299	ADI1	Catalyzes the formation of formate and 2-keto-4- methylthiobutyrate (KMTB) from 1,2-dihydroxy-3-keto-5- methylthiopentene (DHK-MTPene)
Auxin efflux carrier								
FCIRG_12316T1	-2,0638	0.004437	-3,9266	2.95E-07	-1,8628	0.018848	PGUG_01990	auxin efflux carrier
ICSH								
FCIRG_14838T1	-2,9379	0.000859	-4,4853	5.59E-07	0,0000		FG07259.1	Isochorismatase
FCIRG_03234T1	-3,4771	0.000633	-4,4394	1.56E-05	0,0000		FG01746.1	Isochorismatase family hydrolase
