# Supplementary material for: The transcriptome of Pinus pinaster under Fusarium circinatum challenge
Source: BMC Genomics. 2020 Jan 8;21:28. doi: 10.1186/s12864-019-6444-0 (PMC6950806; doi:10.1186/s12864-019-6444-0)
Supplement: Supplementary file 15 — Additional file 15: Fusarium circinatum DE genes related to hormone production with hits in the Pathogen Host Interaction (PHI) database. [file 12864_2019_6444_MOESM15_ESM.pdf]

Additional file 15: *Fusarium circinatum* DE genes related to hormone production with hits in the Pathogen Host Interaction (PHI) database.

Query ID	Gene description	Gene name	PHI accession	Species	% identity	E-value	PHI phenotype
**FCIRG_03234T1**	Isochorismatase family hydrolase (ICSH)	EntB	6565	*Klebsiella pneumoniae*	26.000	2,85E-05	unaffected pathogenicity, reduced virulence
**FCIRG_14291T1**	Catalyzes the formation of formate and 2-keto-4- methylthiobutyrate (KMTB) from 1,2-dihydroxy-3-keto-5-methylthiopentene (DHK-MTPene)	FTF1	5483	*Fusarium oxysporum*	90.909	6,31E-55	reduced virulence
**FCIRG_05399T1**	Gibberellin cluster-C13-oxidase	Akt7	4194	*Alternaria alternata*	22.794	5,95E-06	increased virulence (hypervirulence), reduced virulence
		Cyp51a	2907	*Fusarium graminearum*	25.248	4,55E-05	unaffected pathogenicity, reduced virulence
		Cyp51a	1155	*Zymoseptoria tritici*	22.330	6,82E-04	chemistry target: resistance to chemical
		Hic-15	7173	*Verticillium dahliae*	25.366	8,41E-04	reduced virulence
**FCIRG_05404T1**	Gibberellin cluster-GA14-synthase	o-methylsterigmatocystin_oxidoreductase	2393	*Fusarium graminearum*	27.485	4,39E-10	increased virulence (hypervirulence)
		Mocyp51a	2190	*Magnaporthe oryzae*	23.767	4,16E-07	reduced virulence
		Mocyp51b	2191	*Magnaporthe oryzae*	24.878	5,87E-07	unaffected pathogenicity
		Fgerg5a	3037	*Fusarium graminearum*	27.950	3,90E-06	reduced virulence
		Akt7	4194	*Alternaria alternata*	27.273	1,90E-05	increased virulence

							(hypervirulence), reduced virulence
		Cyp51b	2908	*Fusarium graminearum*	24.752	3,26E-05	unaffected pathogenicity, reduced virulence
		Fgerg5b	3038	*Fusarium graminearum*	29.747	1,09E-04	reduced virulence
		Hic-15	7173	*Verticillium dahliae*	23.529	1,11E-04	reduced virulence
		Bxcyp33c9	4607	*Bursaphelenchus xylophilus*	24.675	1,57E-04	reduced virulence
		C14dm	3258	*Leishmania major*	26.490	2,99E-04	reduced virulence
		Erg11	8030	*Candida albicans*	21.285	5,69E-04	loss of pathogenicity
