# Supplementary material for: The transcriptome of Pinus pinaster under Fusarium circinatum challenge
Source: BMC Genomics. 2020 Jan 8;21:28. doi: 10.1186/s12864-019-6444-0 (PMC6950806; doi:10.1186/s12864-019-6444-0)
Supplement: Supplementary file 16 — Additional file 16:. RNA-seq data statistics for each sample at each time point, before and after filtering and trimming. Dpi: days post-inoculation; BR: biological replicate, RIN: RNA Integrity Number; Q 30: Phred quality score 30. [file 12864_2019_6444_MOESM16_ESM.pdf]

Additional file 16: RNA-seq data statistics for each sample at each time point, before and after filtering and trimming. Dpi: days post-inoculation; BR: biological replicate, RIN: RNA Integrity Number; Q 30: Phred quality score 30.

		Raw data					Trimmed and quality data					
Group	Dpi	BR	RIN	Total read pairs	length	Q 30 (%)	Surviving read pairs		Dropped sequences		length	Q 30 (%)
Mock-inoculated	3	1	9.1	47,560,680	101	95.432	43,524,073	92%	4,036,607	8%	87	100%
		2	9.3	49,304,321	101	93.399	42,888,235	87%	6,416,086	13%	87	100%
		3	9.4	55,202,436	101	95.335	50,469,767	91%	4,732,669	9%	87	100%
		4	9.3	57664110	101	93.799	50,255,464	87%	7,408,646	13%	87	100%
	5	1	9.4	60,020,039	101	95.462	54,989,003	92%	5,031,036	8%	87	100%
		2	9.5	48,698,072	101	94.791	43,962,376	90%	4,735,696	10%	87	100%
		3	9.4	67,646,789	101	95.166	61,685,025	91%	5,961,764	9%	87	100%
		4	9.5	49,237,615	101	94.069	43,128,434	88%	6,109,181	12%	87	100%
	10	1	8.9	76,067,369	101	95.59	71,868,450	94%	4,201,529	6%	87	100%
		2	9.1	86,509,928	101	96.1	82,513,169	95%	3,997,603	5%	87	100%
		3	9.5	90,517,427	101	95.9	86,045,866	95%	4,470,914	5%	87	100%
		4	9.3	40,772,260	101	93.827	35,479,626	87%	5,292,634	13%	87	100%
Inoculated	3	1	9	47,685,626	101	95.324	43,557,040	91%	4,128,586	9%	87	100%
		2	8.7	48,814,763	101	94.603	44,193,721	91%	4,621,042	9%	87	100%
		3	8.4	53,563,502	101	95.721	49,227,053	92%	4,336,449	8%	87	100%
		4	9.3	70,028,248	101	94.056	61,288,640	88%	8,739,608	12%	87	100%
	5	1	9	56,674,997	101	95.408	51,758,117	91%	4,916,880	9%	87	100%
		2	9.1	50,285,308	101	95.160	45,541,827	91%	4,743,481	9%	87	100%
		3	9.3	56,625,000	101	94.341	50,588,768	89%	6,036,232	11%	87	100%
		4	9.4	46,585,164	101	94.170	40,838,549	88%	5,746,615	12%	87	100%
	10	1	7.4	80,561,442	101	93.880	76,283,629	95%	4,278,447	5%	87	100%
		2	8.9	48,417,238	101	95.88	42,289,518	87%	6,127,720	13%	87	100%
		3	5.4	-	-	-	-	-	-	-	-	-
		4	5.8	-	-	-	-	-	-	-	-	-
Total				1,288,442,334			1,172,376,350	91%	116,069,425	9%		
